# Male Rat Model of Chemical Androgen Deprivation and Estrogenization from the Perspective of Anthropometric, Histological, and Biochemical Parameters

**DOI:** 10.3390/medicina62010008

**Published:** 2025-12-19

**Authors:** Pavle Ćosić, Milica Vukojević, Marko Miler, Branko Filipović, Milica Manojlović-Stojanoski, Vladimir Ajdžanović

**Affiliations:** Department of Cytology, Institute for Biological Research “Siniša Stanković”—National Institute of the Republic of Serbia, University of Belgrade, 11060 Belgrade, Serbiamanojlo@ibiss.bg.ac.rs (M.M.-S.);

**Keywords:** androgen deprivation, estrogenization, rat models

## Abstract

*Background and Objectives*: Chemical androgen deprivation and estrogenization are essential components of clinical treatment for advanced prostate cancer and male-to-female sex transition. The aim of this study was to determine the effects of these therapies on anthropometric parameters, liver histology, and biochemical parameters, with the goal of establishing experimental models that accurately represent current clinical practice. *Materials and Methods*: Young adult Wistar rats were divided into nine groups: intact control (**IC**), control vehicle (**CV**), cyproterone acetate-treated (**CA**), flutamide-treated (**F**), control sesame oil (**CO**), estradiol valerate-treated (**E**), combined control (**CC**), flutamide + estradiol valerate (**F + E**), and cyproterone acetate + estradiol valerate (**CA + E**)-treated groups. Treatments were administered by subcutaneous injection for four weeks. *Results*: The administration of estradiol valerate, alone or combined with antiandrogens, reduced final body mass and white adipose tissue mass. Notable changes were observed in absolute and relative pituitary, liver, prostate, and testis mass in the **E**, **F + E** and **CA + E** groups. There were no significant changes in liver histology or glycogen deposition; however, the combined treatment groups showed an increased volume density of binucleated hepatocytes and fibrotic tissue. Regarding biochemical parameters, androgen deprivation and/or estrogenization caused marked changes in serum triglyceride, LDL (low-density lipoproteins), ALP (alkaline phosphatase), AST (aspartate aminotransferase), ALT (alanine aminotransferase), Bil-T (bilirubin), creatinine, and urea levels. *Conclusions*: Given the importance of these therapies in clinical practice, providing a model based on the evaluated parameters offers a solid platform for future research.

## 1. Introduction

Chemical androgen deprivation (castration) represents a provoked reduction in circulating endogenous androgen concentrations and their physiological effects, mainly in male individuals, through the administration of specific chemical compounds [1]. In terms of clinical application, androgen deprivation serves as a critical therapeutic approach for treating advanced prostate cancer in men and is also used as part of sex reassignment therapy to reduce circulating testosterone levels in male-to-female sexual transitions [2,3]. Essentially, there are two types of antiandrogens: steroidal (cyproterone acetate), which not only block the androgen receptor (AR) but also inhibit testosterone synthesis by suppressing luteinizing hormone-releasing hormone (LHRH) production in the hypothalamus; and non-steroidal (bicalutamide, flutamide, nilutamide), which compete with androgens for binding to the AR, thereby inhibiting AR function [4,5]. Estrogenization, broadly defined as the treatment of male subjects with female sex hormones (estrogens), is used in male-to-female transition as a mean of achieving desired female physical characteristics (breast growth, reduction in male-pattern body hair, female-pattern fat redistribution), and also as a classical form of prostate cancer treatment [6,7]. However, each therapeutic approach carries health risks that need to be considered. Chronic estrogen treatment can increase the risk of venous thromboembolism and cardiovascular complications in both settings, while the associated feminizing physical traits are generally undesirable in men undergoing prostate cancer treatment [7,8,9]. Conversely, the use of antiandrogens can also have negative health implications in both therapies, with the most common side effects being hepatotoxicity, gynecomastia (considered undesirable in men not undergoing sex transition), cardiovascular toxicity, nausea, and impotence [10]. Although advances in treatment have led to the use of safer forms of estrogen medication (such as estradiol valerate instead of ethinyl estradiol), risks remain, albeit with lower incidence and severity [11]. Therefore, there is an objective need to re-evaluate the effects of the particular treatments at the experimental level, as recent clinical data on their impact on metabolic and neuroendocrine health, especially during sex reassignment, remain scarce [12,13].

Animal models present an essential component of in vivo biomedical research, and the selection of appropriate models for experimental procedures greatly impacts the validity of results and the translational potential of studies [14]. Naturally, having established experimental models of the aforementioned therapeutic approaches, with well documented outcomes, would prove beneficial for the refinement of future research and clinical practice, considering that the negative effects on health and quality of life still persist, as noticed. Experimental models of male androgen deprivation—castration (a brief summary is presented in Table 1) and estrogenization—have been in use for decades; however they often show poor correspondence with applied clinical therapies, where a significant number of studies retain the use of surgical orchidectomy (castration) as a means of achieving androgen deprivation. Although this method effectively eliminates endogenous androgens in experimental settings, from the perspective of clinical application it is less favorable compared with chemical androgen deprivation, as the latter does not cause physical disfigurement and is conveniently available as long-acting depot injections [15]. In the case of estrogenization, some experiments exploited old-generation and less safe forms of estrogen compounds, while studies employing novel estrogen formulations in rats, particularly males, remain few in numbers. With that in mind, the aim of this study is to provide a foundation for establishing and defining an advanced model of the androgen deprivation and/or estrogenization of male rats, which implies the use of antiandrogen compounds (flutamide or cyproterone acetate) and/or estradiol valerate (for its long-acting estrogenic properties). As our research group has prior experience in the application of the orchidectomy and estrogenization of male rats, both adult and middle-aged, in the field of endocrinological and metabolic research, the potential advancement achieved by this work could serve as a significant improvement for future endeavors [16,17,18,19,20,21].

The effects of the chemical androgen deprivation and/or estrogenization of male rats have been evaluated from the view of essential anthropometric, histopathological, and biochemical parameters in this study. This study will assess mass fluctuation, absolute and relative white adipose tissue (WAT) mass variations, absolute and relative organ mass changes, and calculate the effects of treatments through complex anthropometric parameters. A basic histological evaluation of the liver, reflecting the safety of applied chemical compounds, will be performed using H&E, Periodic acid—Schiff (PAS), and Masson’s trichrome staining, followed by quantification. A biochemical analysis of blood glycemic profile, lipid profile, and markers of liver and kidney status will also be conducted. This will essentially allow the establishment of an enhanced model of androgen deprivation and/or estrogenization and lay a solid foundation for better future research of their effects on endocrinological, metabolic, and cardiovascular health.

## 2. Materials and Methods

### 2.1. Animals and Experimental Design

**Figure 1:**
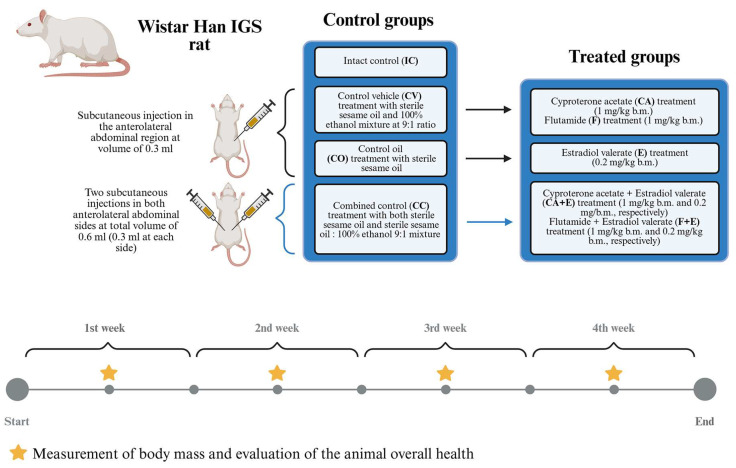
Schematic representation of experimental design.

The experiment involved 45 adult male Wistar Han IGS rats (3 months old at the start of treatment), bred at the Institute for Biological Research “Siniša Stanković “—National Institute of the Republic of Serbia, University of Belgrade, Serbia. The rats were housed 2–3 per cage (polycarbonate cages, 425 mm × 265 mm × 180 mm, ZOONLAB, Castrop-Rauxel, Germany). They were kept at constantly regulated housing conditions (temperature 21–24 °C, 12:12 h light–dark cycle with lights on at 07:00 h and off at 19:00 h, humidity 40–70%, 14–16 air exchanges per hour) with *ad libitum* access to food (chemical composition of the food was previously described) and water [22]. Animals were divided into 9 groups (5 animals *per* group), with each treated group assigned a corresponding control to correlate with the type of medium the particular treating substance was dissolved in. Keeping that in mind, the rats were placed in the following groups: intact control (**IC**); control vehicle (sterile sesame oil (Granum^®^, Hajdukovo, Serbia) and 100% ethanol (ZORKA Pharma-Hemija d.o.o, 56/230, Šabac, Serbia) mixture 9:1—**CV**) serving as control for cyproterone acetate (MedChemExpress, Lot 13789, Cat. #HY-13604/CS-2891, Monmouth Junction, NJ, USA; 1 mg/kg b.m. dissolved in the sesame oil/100% ethanol 9:1 mixture—**CA**) and flutamide (Sigma-Aldrich, Lot 069K1581, F9397, St. Louis, MO, USA; 1 mg/kg b.m. dissolved in the sesame oil/100% ethanol 9:1 mixture—**F**) treated groups; control oil (sterile sesame oil—**CO**), serving as control for the estradiol valerate group (Sigma-Aldrich, Lot 87H0141, E-1631, St. Louis, MO, USA; 0.2 mg/kg b.m. dissolved in sterile sesame oil—**E**); combined control (received both sterile sesame oil and sterile sesame oil/100% ethanol 9:1 mixture—**CC**) serving as control for flutamide + estradiol valerate (1 mg/kg b.m. + 0.2 mg/kg b.m., **F + E**, respectively) and cyproterone acetate + estradiol valerate (1 mg/kg b.m. + 0.2 mg/kg b.m., **CA + E**, respectively) groups. A schematic representation of the experimental design is shown in Figure 1. Sesame oil was sterilized by method of staged dry sterilization (dry sterilizer (Sutjeska, Belgrade, Serbia), first at 70 °C for 15 min, then at 100 °C for 15 min, and finally at 160 °C for 2 h, with each period measured after reaching the required temperature. The regime of each treatment was designed to mimic existing human clinical treatments: **F** and **CA** groups simulate the therapeutic approach to prostate cancer, while **F + E** and **CA + E** groups simulate hormone replacement therapy during sex reassignment [10,23,24]. Treatment with estradiol valerate alone (**E**) can be considered applicable in both of the above clinical situations (classic hormonal monotherapy for prostate cancer as well as for sex reassignment) [25]. As such, the doses were calculated for rats from exploited human doses using allometric calculation [26].

$$AED(mg/kg)=Human\;dose(mg/kg)\times K_{m}ratio$$

*AED*—animal equivalent dose.

The *K_m_* ratios, which are based on body mass and body surface area, were obtained from the Food and Drugs Administration’s Guidance for Industry on estimating safe starting doses [27].

Dissolved treatments (treated groups) or dissolvent mixtures/dissolvent alone (control groups) were administered via subcutaneous injection in the anterolateral abdominal region at a volume of 0.3 mL, while the **CC**, **F + E**, and **CA + E** groups received two injections (2 × 0.3 mL; appropriate dissolved treatment and the corresponding dissolvent, in both anterolateral abdominal sides) daily for 4 weeks. Once a week, tests were conducted on the overall appearance and general health of each rat according to the detailed questionnaire (Supplementary S1). All efforts were focused to ensure minimal stress and suffering of experimental animals during the experiment. After 4 weeks of treatment, all rats were sacrificed via decapitation 24h after the last injection, and their blood from the trunk and organs was sampled for biochemical and histological analysis. Euthanasia was performed from morning to early afternoon period, approximately between 09:00 h and 14:00 h, and there was no report of fasting period as the animals had *ad libitum* access to food and water until that moment. There were two reported spontaneous deaths of experimental rats, one in the **F** group and one in the **F + E** group at the end of the experiment. In the case of the **F** group animal, autopsy results showed several times enlarged adrenal glands, greenish adipose tissue, and hemorrhagic lungs. The animal from **F + E** group died spontaneously shortly before being sacrificed, and autopsy was not performed; however, there were no visible indicators that suggested poor animal health until that point (see Supplementary S1). Neither animal was included in the statistical analysis. All experimental procedures were conducted in compliance with Directive 2010/63/EU on the protection of animals used for experimental and other scientific purposes and were also approved by the Ethics Committee for the Use of Laboratory Animals of the Institute for Biological Research “Siniša Stanković”—National Institute of the Republic of Serbia, University of Belgrade (Approval No. 01/3207).

### 2.2. Anthropometric Measurements

The initial, simple anthropometric parameters that were measured included body mass (measured once a week during the treatment), nose-to-anus (full body) length, gonadal white adipose tissue (gWAT) mass, inguinal white adipose tissue (iWAT) mass, peritoneal white adipose tissue (pWAT) mass, and total white adipose tissue (totalWAT) mass. Measurements of full body length were taken after decapitation (to avoid stress and the impact of anesthesia on hormonal status), measuring the head and body length (without the tail) separately using a ruler. The specific types of adipose tissue were extracted and weighed in order to determine body composition and body mass gain during the experiment. Complex, derivative parameters (equations for each parameter are provided in Table 2), including body mass index (BMI, g/cm^2^), Lee’s index (g/cm), adiposity index (n.a.), and specific rate of body mass gain (g/kg), were calculated from the aforementioned, initial anthropometric parameter values.

### 2.3. Organ Processing, Histochemistry, and Light Microscopy

Organs were excised, stripped of fat and connective tissue, and placed in fixatives. The left adrenal gland, pituitary, thyroid gland, and prostate were placed in Bouin’s fixative (75 mL 35% formalin stock, 25 mL saturated aqueous solution of picric acid, 5 mL glacial acetic acid; appropriate for fixation of small samples and organs in endocrinological research), whereas the left kidney, a randomly chosen testicle, and a section of peritoneal WAT were placed in 10% formalin. Immediately after removal, a photograph of the whole liver of each animal was taken (Huawei P30 Lite vMAR-LX1A, Triple camera 48MP 1:1.8/27 ASPH, 1× magnification, Huawei Technologies Co., Ltd., Shenzhen, Guangdong, China) for macroscopic evaluation, after which a section of liver tissue was taken and placed in 10% formalin for histological evaluation. After 48 h of fixation, endocrine glands and organs were dehydrated in a series of increasing concentrations of ethanol (30–100%, ZORKA Pharma-Hemija d.o.o, Šabac, Serbia), cleared in xylol (ZORKA Pharma-Hemija d.o.o, 14958/117, Šabac, Serbia), and finally embedded in BIOWAX PLUS 56/58 (BIOGNOST^®^, BioGnost Ltd., Medjugorska 59, 10040 Zagreb, Croatia) ultrafiltered paraffin with added plastic polymers for infiltration and embedding. Liver tissue sections (5 µm thick) were obtained using a rotational microtome (RM 2125RT Leica Microsystems, Wetzlar, Germany) equipped with a high-profile microtome blade (SAKURA Accu-Edge^®^, 4685 routine section, Feather Safety Razor Co., Ltd., Osaka, Japan), placed on the microscopic slides (Epredia, LOT 04324, Portsmouth, NH, USA) and later deparaffinized in xylol (ZORKA Pharma-Hemija d.o.o, 14958/117, Šabac, Serbia) and rehydrated in a series of decreasing concentrations of ethanol (100–70%; ZORKA Pharma-Hemija d.o.o, Šabac, Serbia) in preparation for histochemical staining. For qualitative histology, Hematoxylin & Eosin (H&E), Masson’s trichrome, and Periodic acid-Schiff (PAS) stainings were used. Finally, after all stainings, the coverslips (Menzel-Gläser, Gerhard Menzel, Glasbearbeitungswerk GmbH & Co., Ltd., KG, Braunschweig, Germany) were mounted with DPX (Sigma-Aldrich, Co., Ltd., 06522, St. Louis, MO, USA).

A detailed protocol for Masson’s trichrome staining was previously described [28]. In brief, 5 μm thick liver sections were deparaffinized and incubated in Weigert’s Hematoxylin for 10 min. After rinsing in tap water, the sections were incubated in a mixture of 1% acid fuchsine and 1% ponceau dexylidine (1:2) for 5 min, which were then, after rinsing in several changes of distilled water, incubated in 0.05% phosphomolybdic acid for 10 min, followed by immediate incubation (no rinsing) in 2.5% aniline blue for 5 min. The final steps included rehydration and mounting with DPX (Sigma-Aldrich, Co., Ltd., 06522, St. Louis, MO, USA).

PAS staining was used to visualize glycogen deposits in liver sections. The procedure involved deparaffinization and rehydration (100–70% ethanol, then distilled water; 5 min each step) of tissue sections, which were immediately after incubated in 0.5% periodic acid (Thermo Fisher Scientific, Lot: 102391128, Cat. No. B20433.18, Waltham, MA, USA) for 30 min and subsequently rinsed in distilled water for 5 min. After that, the liver sections were incubated in Schiff’s reagent (prepared from potassium metabisulfite (K_2_S_2_O_5_)—Zorka, Šabac, Serbia; basic fuchsine—Jugolek, 562/9/00, Belgrade, Serbia; activated charcoal—Avena lab, Formadria d.o.o., Ser. No. F241/19, Vršac, Serbia) for 30 min and then rinsed in warm water for 10 min. The next step was nuclear counterstaining with Hematoxylin (Hematoxylin (Sigma-Aldrich, Lot#MKBZ9780V, St. Louis, MO, USA; 100% ethanol (ZORKA Pharma-Hemija d.o.o, 56/230, Šabac, Serbia); potassium aluminum sulfate dodecahydrate (KAl(SO_4_)_2_ × 12H_2_O, ANALYTIKA^®^, Ltd., Prague, Czech Republic))) for 10 s, followed by rinsing in tap water for 5 min. Dehydration in ethanol of increasing concentration (70–100%; 5 min each step), clearing in xylol (2 × 5 min), and mounting in DPX (Sigma-Aldrich, Co., Ltd., 06522, St. Louis, MO, USA) were the final steps of the procedure.

Digital images of stained liver sections were captured using a LEITZ DM RB light microscope (Leica Mikroskopie & Systems GmbH, Wetzlar, Germany; objective magnification 20×), a LEICA DFC320 CCD camera (Leica Microsystems Ltd., Heerbrugg, Switzerland), and the Leica DFC Twain Software (version 4.14.0, Leica, Wetzlar, Germany).

### 2.4. Quantification of Binuclear Cell and Collagen Volume Density, and PAS Staining Intensity

#### 2.4.1. Volume Density Estimation

The volume density (V_V_, %) of binuclear hepatocytes and connective tissue was estimated using a microscope (Olympus BX-51, Evident Corporation, Tokyo, Japan) equipped with a CCD camera (PixelLink, Ottawa, ON, Canada) connected to a 19” PC monitor (Dell, Round Rock, TX, USA). Stage movement control and interactive test grids (uniformly spaced point grids and an unbiased dissector frame) were provided using the newCAST stereology software package (Visiopharm Integrator System, version 2.12.1.0; Visiopharm, Denmark) running on a Dell workstation.

Two central paraffin sections were analyzed per animal, and 20% of the liver section area was uniformly randomly sampled by the software. Five animals were analyzed per group. Test points hitting binuclear hepatocytes (H&E staining) and blue-stained connective tissue (Masson’s trichrome staining) were counted. The volume density (*V_V_*) was calculated as the ratio of the number of points hitting the structure of interest (*P_p_*) to the total number of points hitting the reference space (*P_t_*):

$$V_{V}=(P_{p}/P_{t})\times 100$$

Volume densities of binuclear hepatocytes and connective tissue were calculated for each experimental group.

#### 2.4.2. PAS Staining Intensity Calculation

Glycogen staining intensity was quantified from PAS-stained liver sections using FIJI/ImageJ software (ImageJ version 1.51j8, National Institutes of Health, Bethesda, MD, USA). Each micrograph was subjected to color deconvolution using the “PAS” vector to separate the PAS-positive (magenta) channel from the Hematoxylin counterstain. Regions of interest (ROIs) were defined as segments of hepatocyte cytoplasm within each micrograph. The positive signal was isolated by thresholding, and relative staining intensity was quantified as the mean pixel intensity of ROIs. All images were acquired under 40× magnification and the same acquisition settings.

### 2.5. Biochemical Analyses

Biochemical analyses were performed using the BS-240 Vet Chemistry Analyzer (Shenzen Mindray Animal Medical Technology Co., Ltd., Shenzhen, China). Blood was collected from the trunk and serum samples were stored at −70 °C until the time of analysis. Serum concentrations of the following biochemical parameters were evaluated: glucose (glucose oxidase/peroxidase, BioSystems COD 11503, BioSystems S. A., Barcelona, Spain); triglycerides (glycerol phosphate oxidase/peroxidase, BioSystems COD 11528, BioSystems S. A., Barcelona, Spain); high-density lipoproteins (HDLs) (direct detergent, BioSystems COD 11557, BioSystems S. A., Barcelona, Spain); low-density lipoproteins (LDLs) (detergent, BioSystems COD 11585, BioSystems S. A., Barcelona, Spain); alkaline phosphatase (ALP) (2-amino-2-methyl-1-propanol buffer (IFCC), BioSystems COD 11593, BioSystems S. A., Barcelona, Spain); aspartate aminotransferase (AST) (AST/GOT, BioSystems COD 11531, BioSystems S. A., Barcelona, Spain); alanine aminotransferase (ALT) (ALT/GPT, BioSystems COD 11533, BioSystems S. A., Barcelona, Spain); bilirubin (Bil-T) (diazotized sulfanilic, BioSystems COD 11510, BioSystems S. A., Barcelona, Spain); gamma-glutamil transferase (GGT) (BioSystems COD 11520, BioSystems S. A., Barcelona, Spain); creatinine (Jaffé, BioSystems COD 11502, BioSystems S. A., Barcelona, Spain); and urea (urease/glutamate dehydrogenase, BioSystems COD 11516, BioSystems S. A., Barcelona, Spain). Detailed procedures for biochemical analyses are available in Supplementary S3.

### 2.6. Statistical Analysis

The experimental design is based on the assumption that changes in morphofunctional parameters represent the primary endpoints to be measured. Sample size estimation was performed using G*Power software (version 3.1.9.7; Düsseldorf, Germany). Based on previously published data [18], the expected effect size is medium (Cohen’s *d* = 0.5), with an estimated standard deviation of *s* = 1. This calculation yielded a total required sample size of 45 animals, with *n* = 5 animals *per* experimental group, providing a statistical power of 80% to detect significant differences in the selected study outcomes. Analysis of numerical data was performed using GraphPad Prism v8.0.2 software (GraphPad Software, Boston, MA, USA). One-way ANOVA was used to determine the differences between experimental groups, except when evaluating differences in fluctuation of body mass within groups during the experiment, in which case two-way ANOVA was used, followed by Dunnett’s post hoc test. Brown–Forsythe’s and Bartlett’s tests were used to determine the equality of variances between groups. Statistical significance was considered as * *p* < 0.05, ** *p* < 0.01, *** *p* < 0.001, and **** *p* < 0.0001 levels of significance. All values are presented as group mean ± standard deviation (SD).

## 3. Results

### 3.1. Final Body Mass, Full Body Length, Absolute and Relative White Adipose Tissue (WAT) Mass, and Body Mass Fluctuation

The final body mass, nose-to-anus (full body) length, and absolute and relative WAT (gWAT, iWAT, pWAT, and totalWAT) mass of the animals used in the experiment are shown in Table 3. The final body mass of rats treated with estradiol valerate (**E**) decreased by 28.64% (*p* < 0.001) in comparison with the control oil group (**CO**). In animals treated with cyproterone acetate + estradiol valerate (**CA + E**) final body mass decreased by 31.53% (*p* < 0.0001), while flutamide + estradiol valerate (**F + E**) combined treatment decreased rat final body mass by 30.83% (*p* < 0.0001), all compared with combined control (**CC**) animals. The treatment of experimental rats with estradiol valerate (**E**) caused a decrease in full body length by 11.54% (*p* < 0.01) when compared with the control oil (**CO**) group, while **E** combinations with cyproterone acetate (**CA + E**) and flutamide (**F + E**) also caused a decrease in the mentioned parameter by 13.6% (*p* < 0.001) and 12.8% (*p* < 0.001), respectively, in comparison with the combined control (**CC**). Absolute gWAT mass was reduced by 23.49% (*p* < 0.05) and 19.46% (*p* < 0.05) in the control vehicle (**CV**) and combined control (**CC**) group, respectively, compared with the intact control (**IC**). On the other hand, the treatment of male rats with cyproterone acetate (**CA**) increased absolute gWAT mass by 51.86% (*p* < 0.001) in comparison with **CV** males. A reduction in gWAT mass by 50.84% (*p* < 0.001) was found after the estradiol valerate (**E**) treatment of adult males, in comparison with control oil (**CO**) rats. Treatment with a combination of cyproterone acetate and estradiol valerate (**CA + E**) caused a decrease in gWAT mass by 40.37% (*p* < 0.001), while treatment with flutamide and estradiol valerate (**F + E**) caused a decrease in the same parameter by 32.51% (*p* < 0.01), all when compared with the **CC** group. Treatment with estradiol valerate (**E**) reduced pWAT mass by 36.01% (*p* < 0.05), as well as totalWAT mass by 38.54% (*p* < 0.05), all in comparison with the corresponding **CO** control values. However, relative gWAT mass was increased by 44.6% (*p* < 0.01) in the **CA** group compared with **CV** rats, while treatment with flutamide and estradiol valerate (**F + E**) caused an increase in relative iWAT mass by 77.42% (*p* < 0.05) compared with the combined control (**CC**) group.

**Figure 2:**
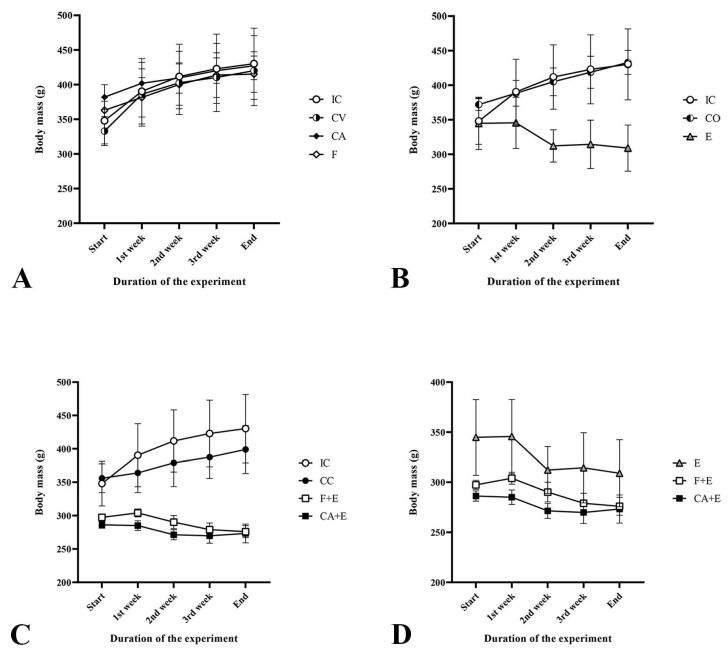
Fluctuations of the rat body masses during the experiment: (**A**) chemical androgen deprivation, (**B**) estrogenization, (**C**) combined chemical androgen deprivation and estrogenization, (**D**) and all together, treatments compared to each other. Groups are marked with initials indicating the adequate control or treatment: **IC**—intact control, **CV**—control vehicle, **CA**—cyproterone acetate, **F**—flutamide, **CO**—control sesame oil, **E**—estradiol valerate, **CC**—combined control, **F + E**—flutamide + estradiol valerate, **CA + E**—cyproterone acetate + estradiol valerate group of rats. All values are presented as mean ± SD, n = 5 animals *per* group.

The fluctuation in body mass during the experiment is presented in Figure 2. Control groups all show a similar trend of increase in body mass during the duration of experiments, with a steep rise after the first and second weeks, reaching a plateau towards the end of the experiment. The combined control group (**CC**) shows a less pronounced trend of body mass increase, with a gentle, steady slope compared with the other controls. Evidently, the treatment of rats with estradiol valerate (**E**) caused a decrease in body mass, with the largest drop recorded during the second week of treatment, after which the decrease reached almost a plateau until the end of the experiment. Similarly, the treatment of rats with the combination of cyproterone acetate and estradiol valerate (**CA + E**), as well as the treatment with flutamide and estradiol valerate (**F + E**), resulted in a reduction in body mass, with the largest decrease observed during the second week of treatment, although this was not as pronounced as in the **E** group.

### 3.2. Complex Anthropometric Parameters—Body Mass Index (BMI), Lee’s Index, Adiposity Index, and Specific Rate of Body Mass Gain

**Figure 3:**
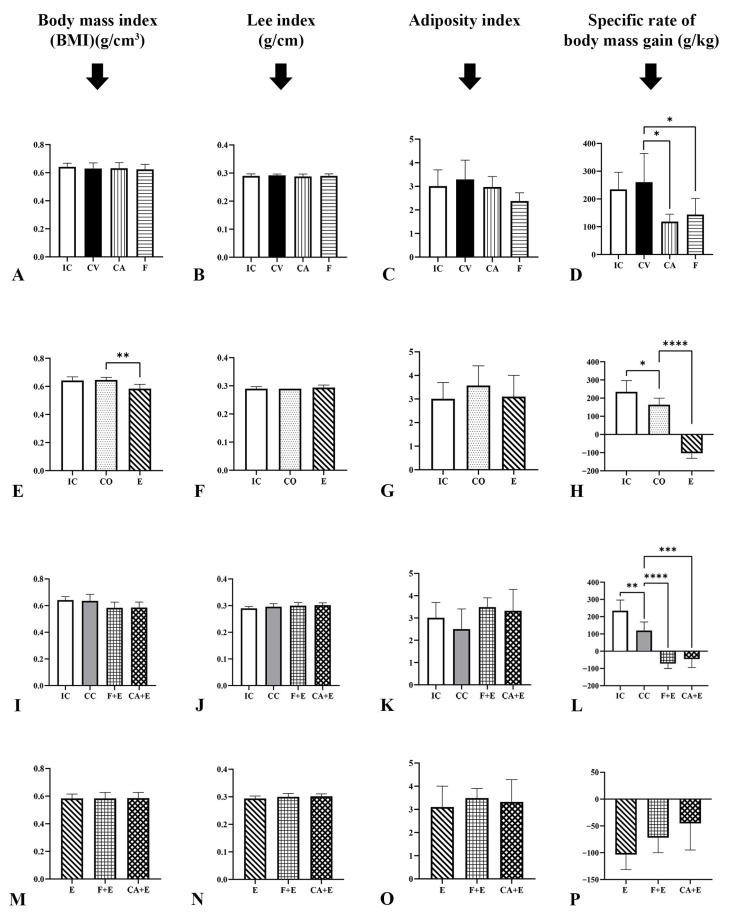
Changes in the complex anthropometric parameters (BMI, Lee’s index, adiposity index, specific rate of body mass gain) of the experimental rats: chemical androgen deprivation (**A**–**D**), estrogenization (**E**–**H**), combined chemical androgen deprivation and estrogenization (**I**–**L**), and all together, treatments compared to each other (**M**–**P**). **IC**—intact control, **CV**—control vehicle, **CA**—cyproterone acetate, **F**—flutamide, **CO**—control sesame oil, **E**—estradiol valerate, **CC**—combined control, **F + E**—flutamide + estradiol valerate, **CA + E**—cyproteorne acetate + estradiol valerate group of rats. All values are presented as mean ± SD, n = 5 animals *per* group. * *p* < 0.05, ** *p* < 0.01, *** *p* < 0.001, **** *p* < 0.0001.

A graphical representation of the effects of treatments on the body mass index (BMI), Lee’s index, adiposity index, and specific rate of body mass gain can be found in Figure 3. After the treatment of rats with estradiol valerate (**E**) BMI was reduced by 9.6% (*p* < 0.01) in comparison with the same parameter in the **CO** group. There were no significant changes in the Lee index or the adiposity index after any treatment. The control oil (**CO**) and combined control (**CC**) group showed a decrease in the specific rate of body mass gain by 30.17% (*p* < 0.05) and 48.96% (*p* < 0.01), respectively, compared with intact control (**IC**) rats. The specific rate of body mass gain was reduced after the cyproterone acetate (**CA**) and flutamide (**F**) treatment of male rats by 54.45% (*p* < 0.05) and 44.82% (*p* < 0.05), respectively, in comparison with control vehicle (**CV**) animals. Estradiol valerate (**E**) administered to rats caused a decrease in the specific rate of body mass gain by 163.1% (*p* < 0.0001) compared with the **CO** group. The administration of cyproterone acetate and estradiol valerate (**CA + E**) to rats reduced the body mass gain rate by 137.81% (*p* < 0.001) when compared with **CC** animals, while the administration of flutamide with estradiol valerate (**F + E**) caused an even greater reduction in the specific rate of body mass gain by 160.04% (*p* < 0.0001) in comparison to the same combined control (**CC**) group.

### 3.3. Absolute Organ Mass

Absolute organ mass data is presented in Table 4. Treatment with flutamide (**F**) or cyproterone acetate (**CA**) had no significant impact on pituitary gland mass. Estradiol valerate (**E**) caused a marked increase in the pituitary mass of male rats by 3.61 times (3.61×) (*p* < 0.0001) in comparison with the **CO** group. The combination of flutamide and estradiol valerate (**F + E**) treatment caused an increase in the pituitary mass of rats by 3.57-fold (3.57×) (*p* < 0.0001) compared with **CC** animals, while in the case of the cyproterone acetate and estradiol (**CA + E**) combination the increase in the same parameter was 2.91-fold (2.91×) (*p* < 0.001) compared with the **CC** group. Absolute left adrenal gland mass was increased only in the **E** group by 17.65% (*p* < 0.05), in comparison with the control oil (**CO**) group. After the administration of sterile sesame oil (**CO**) to rats, absolute liver mass was increased by 11.25% (*p* < 0.05) compared with intact control rats (**IC**). Treatment with estradiol valerate (**E**) decreased absolute liver mass by 33.61% (*p* < 0.0001) in comparison with the **CO** corresponding values. Animals that received flutamide and estradiol valerate (**F + E**), as well as cyproterone acetate and estradiol valerate (**CA + E**), showed a reduction in absolute liver mass by 24.29% (*p* < 0.01) and 19.64% (*p* < 0.05), respectively, in comparison with **CC** rats. Absolute mass of the left kidney was found to be decreased only in the **F + E** group of animals by 25.41% (*p* < 0.05) compared with the combined control (**CC**) group. The estradiol valerate (**E**) treatment of adult male rats reduced absolute prostate mass by 91.17% (*p* < 0.0001) compared with control oil (**CO**) adult males. Similarly, the combined administration of flutamide and estradiol valerate (**F + E**) caused a decrease in absolute prostate mass by 90.5% (*p* < 0.0001) compared with the **CC** group, while treatment with cyproterone acetate and estradiol valerate (**CA + E**) caused a decrease in absolute prostate mass by 91.3% (*p* < 0.0001), also compared with the **CC** group. Absolute heart mass was reduced in the **E** group by 23.72% (*p* < 0.01) compared with control oil (**CO**) male rats. In the **F + E** group, absolute heart mass was decreased by 38.01% (*p* < 0.001) compared with the combined control (**CC**) group, while in the **CA + E** group, absolute heart mass was decreased by 34.82% (*p* < 0.001) compared with **CC** rats. The combined control (**CC**) group showed a decrease in absolute testis mass of 15.02% (*p* < 0.05) compared with intact control (**IC**) rats. The treatment of rats with estradiol valerate (**E**) decreased absolute testis mass by 83.74% (*p* < 0.0001) compared with the **CO** group. The combined administration of flutamide and estradiol valerate (**F + E**) caused a decrease in testis mass by 83.58% (*p* < 0.0001) compared with combined control (**CC**) animals, as well as of 19.2% (*p* < 0.05) when compared with rats treated with estradiol valerate (**E**) alone. Absolute testis mass in the **CA + E** group was decreased by 80.7% (*p* < 0.0001) in comparison with the **CC** group of animals.

### 3.4. Relative Organ Mass

Relative organ mass data is presented in Table 5. Relative pituitary mass was increased by 5.16 times (5.16×) (*p* < 0.0001) in the **E** group of males compared with **CO** controls. The combined administration of flutamide and estradiol valerate (**F + E**) caused an increase in relative pituitary mass by 5.23 times (5.23×) (*p* < 0.0001) compared with **CC** male rats. The cyproterone acetate and estradiol valerate (**CA + E**) combined treatment increased relative pituitary mass by 4.26-fold (4.26×) (*p* < 0.001) in comparison with the combined control (**CC**). In adult male rats treated with flutamide (**F**), relative adrenal gland mass increased by 21.15% (*p* < 0.01), compared with the control vehicle (**CV**) group of rats. The estradiol valerate (**E**) treatment of rats increased relative adrenal mass by 64.78% (*p* < 0.0001) in comparison with the **CO** group. Combined treatment with cyproterone acetate and estradiol valerate (**CA + E**) caused an increase in relative adrenal mass by 40.46% (*p* < 0.01) compared with the combined control (**CC**). The administration of sterile sesame oil and 100% ethanol mixture (**CV**) caused an increase in relative liver mass by 17.45% (*p* < 0.05) compared with the intact control (**IC**) values. In the **CO**, **CC**, **F**, **CA,** and **E** groups there were no significant changes in relative liver mass. On the other hand, groups of combined treatment regimes **F + E** and **CA + E** showed an increase in relative liver mass of 17% (*p* < 0.05) and 24.33% (*p* < 0.01), respectively, compared with treatment with estradiol valerate (**E**) alone, while in the **CA + E** group there was also an increase in the aforementioned parameter of 17.75% (*p* < 0.01) when compared with combined control (**CC**) rats. Relative left kidney mass increased by 20.37% (*p* < 0.01) in the **E** group compared with the **CO** group, while in the **CA + E** group the same parameter increased by 15.96% (*p* < 0.05) compared with the **CC** group. A significant decrease in relative prostate mass (87.42% (*p* < 0.0001)) was observable after the treatment of male rats with estradiol valerate (**E**) compared with control oil (**CO**) values. Similarly, combined treatments with flutamide and estradiol valerate (**F + E**) as well as with cyproterone acetate and estradiol valerate (**CA + E**) decreased relative prostate mass by 86.35% (*p* < 0.0001) and 87.34% (*p* < 0.0001), respectively, in comparison with the combined control (**CC**) group. There were no significant changes in relative heart mass values after treatments of adult male rats. Relative testis mass decreased by 77.14% (*p* < 0.0001) after treatment with estradiol valerate (**E**) compared with **CO** rats. The administration of cyproterone acetate or flutamide with estradiol valerate (**CA + E**, **F + E**) caused a decrease in relative testis mass by 72.04% (*p* < 0.0001) and 76.37% (*p* < 0.0001), respectively, in comparison with the combined control (**CC**) group.

### 3.5. Liver Macroscopic, Qualitative, and Quantitative Histological Evaluation

**Figure 4:**
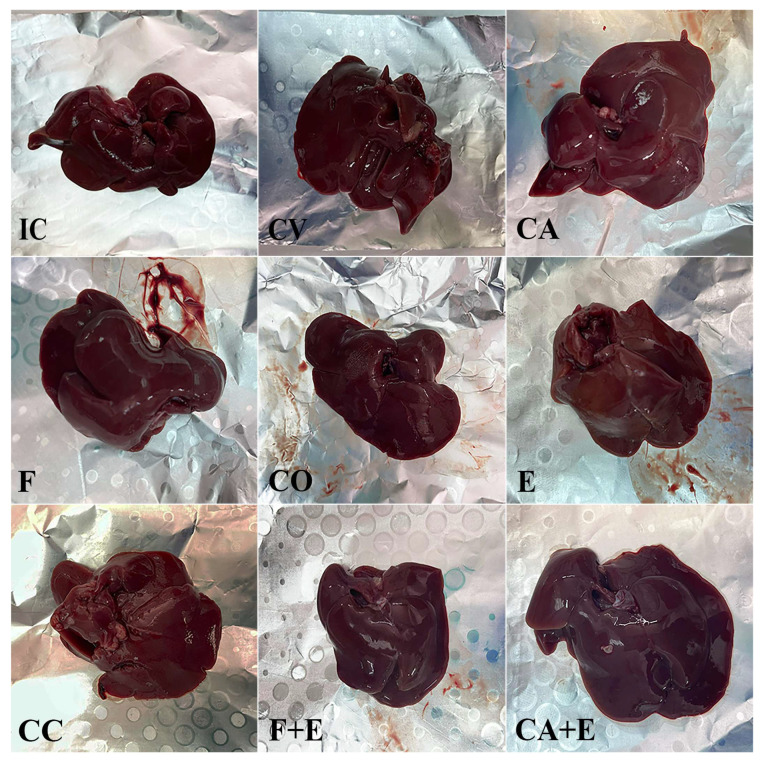
Macroscopic examination of the liver in the control and experimental groups of rats. Groups are marked with initials indicating the adequate control or treatment: **IC**—intact control, **CV**—control vehicle, **CA**—cyproterone acetate, **F**—flutamide, **CO**—control sesame oil, **E**—estradiol valerate, **CC**—combined control, **F + E**—flutamide + estradiol valerate, **CA + E**—cyproterone acetate + estradiol valerate group of rats.

**Figure 5:**
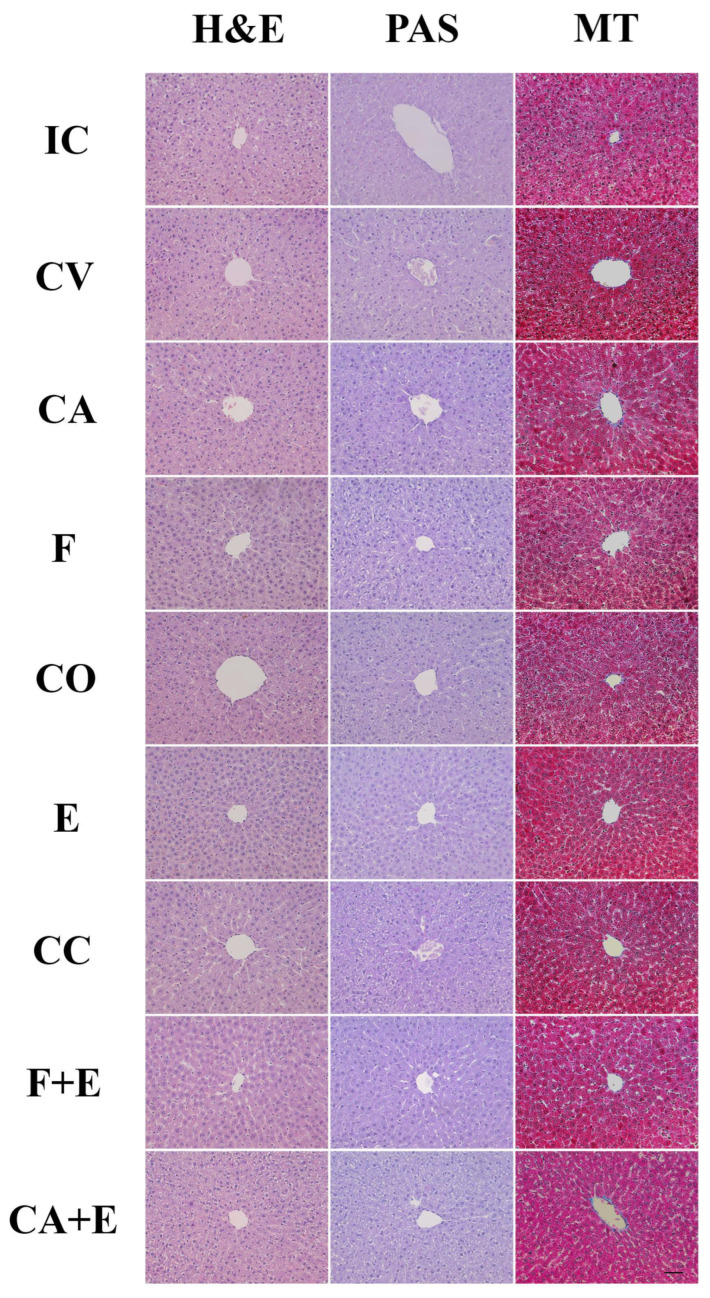
Liver histology in the following: **IC**—intact control, **CV**—control vehicle, **F**—flutamide, **CA**—cyproterone acetate, **CO**—control sesame oil, **E**—estradiol valerate, **CC**—combined control, **F + E**—flutamide + estradiol valerate, **CA + E**—cyproterone acetate + estradiol valerate group of rats. Histochemical H&E, Masson’s trichrome (MT), and PAS staining. Scale bar: 100 µm. Objective magnification 20×, scale bar 50 µm.

**Figure 6:**
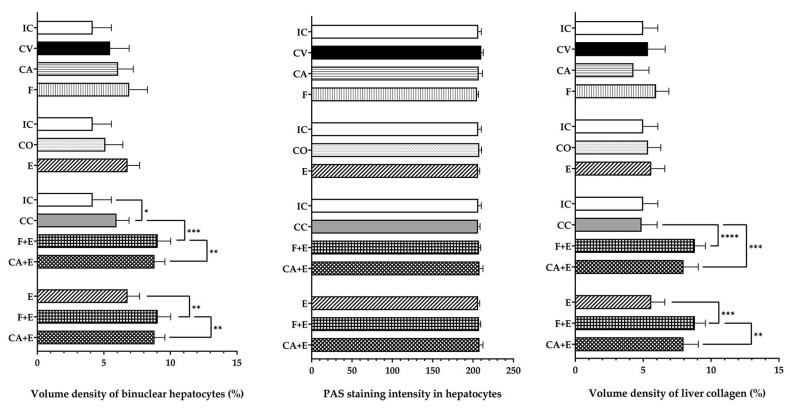
Changes in binuclear hepatocyte volume density (%), PAS staining intensity, and collagen volume density (%) in the following: **IC**—intact control, **CV**—control vehicle, **F**—flutamide, **CA**—cyproterone acetate, **CO**—control sesame oil, **E**—estradiol valerate, **CC**—combined control, **F + E**—flutamide + estradiol valerate, **CA + E**—cyproterone acetate + estradiol valerate group of rats. All values are presented as mean ± SD, n = 5 animals *per* group. * *p* < 0.05, ** *p* < 0.01, *** *p* < 0.001, **** *p* < 0.0001.

Macroscopic liver images and photomicrographs of liver histological sections are presented in Figure 4 and Figure 5, respectively. The livers of the control animals are characterized by four lobes of different sizes, a characteristic reddish-brown color, smooth surface, no lesions, and a firm but pliable consistency. Macroscopic evaluation showed no changes in liver surface morphology or tissue integrity after the treatments; the liver appeared healthy. A qualitative histological analysis of liver sections was performed using H&E, PAS, and Masson’s trichrome staining. In control animals, the liver lobule represents the basic structural and functional unit, with the **central vein** located in the center of each lobule and **portal triads** found at the corners, which can be clearly observed. The hepatocytes extend radially from the central vein and make up the largest part of the liver parenchyma. They are polygonal with a centrally located nucleus, and some hepatocytes are binucleated. The analysis showed a normal liver parenchyma and arrangement of hepatocytes, with no evidence of significant pathological changes (necrosis and inflammation) in most cases (experimental groups), although increased fibrosis and more frequent binuclear cells were generally observed, particularly in the **CA + E** and **F + E** groups. Quantitative histological analysis (presented in Figure 6) showed an increased volume density of binuclear hepatocytes in the **CC** group compared with the **IC** group by 42.84% (*p* < 0.05). The treatment of male rats with a combination of flutamide and estradiol valerate increased this parameter by 52.02% (*p* < 0.001), while combined treatment with cyproterone acetate and estradiol valerate increased it by 48.02% (*p* < 0.01), both compared with combined control animals (**CC**). The **F + E** and **CA + E** groups showed increased binuclear hepatocyte volume density by 33.33% (*p* < 0.01) and 29.83% (*p* < 0.01), respectively, compared with the **E** group. The evaluation of PAS staining intensity showed no significant change in this parameter between experimental groups. In contrast, collagen volume density was significantly increased after the administration of flutamide and estradiol valerate to male rats by 80.50% (*p* < 0.0001) compared with the combined control group (**CC**), while the administration of cyproterone acetate and estradiol valerate increased the same parameter by 60.41% (*p* < 0.001), also compared with the combined control group (**CC**). Compared with treatment with estradiol valerate alone (**E**), combination treatment with flutamide and cyproterone acetate (**F + E** and **CA + E** groups) increased the volume density of liver collagen by 57.56% (*p* < 0.001) and 42.56% (*p* < 0.01), respectively.

### 3.6. Biochemical Parameters

Values of the glucose and lipid profiles in control and treated groups are presented in Table 6. The treatments did not significantly affect blood glucose concentration. The administration of a sterile sesame oil/100% ethanol mixture (**CV**) (ratio 9:1) caused an increase in the serum level of triglycerides by 55.06% (*p* < 0.05) compared with the intact control (**IC**) group. The combined treatment of male rats with cyproterone acetate and estradiol valerate (**CA + E**), as well as with flutamide and estradiol valerate (**F + E**), resulted in a marked increase in blood triglyceride levels by 107.97% (*p* < 0.001) and 95.65% (*p* < 0.01), respectively, compared with the **CC** group. Serum HDL concentration was significantly increased only in the **CA + E** group by 30.65% (*p* < 0.01) compared with the combined control (**CC**) group. In the **CC** group, serum LDL concentration was increased by 50% (*p* < 0.01) compared with the **IC** group. Estradiol valerate (**E**) was shown to decrease blood LDL level by 47.5% (*p* < 0.01) compared with rats that received sterile sesame oil (**CO**). The treatment of rats with flutamide and estradiol valerate (**F + E**) caused a decrease in blood LDL level by 79.17% (*p* < 0.0001) compared with **CC** rats and by 40.48% (*p* < 0.01) compared with rats treated with estradiol valerate (**E**) alone. A similar effect was observed in the **CA + E** group, where serum LDL concentration was decreased by 79.17% (*p* < 0.0001) compared with the **CC** group and by 40.48% (*p* < 0.01) compared with the **E** group (Table 6).

Values of the hepatorenal biochemical profile in control and treated groups are presented in Table 7. The cyproterone acetate (**CA**) treatment of rats caused a significant increase in serum ALP concentration by 93.13% (*p* < 0.001) compared with the control vehicle (**CV**) group. On the other hand, the administration of estradiol valerate (**E**) caused a decrease in blood ALP level by 54.02% (*p* < 0.001) compared with the control oil (**CO**) group of rats. After combined treatment with cyproterone acetate and estradiol valerate (**CA + E**), the concentration of serum ALP was decreased by 39.29% (*p* < 0.01) compared with combined control (**CC**) values. In the **F + E** group, serum AST level was decreased by 29.53% (*p* < 0.05) compared with the **E** group, while in the **CA + E** group the same parameter was lowered by 27.85% (*p* < 0.05) and 40.98% (*p* < 0.01) compared with the **CC** and **E** groups, respectively. The administration of flutamide and estradiol valerate (**F + E**) reduced ALT levels by 33.03% (*p* < 0.05), while the administration of cyproterone acetate and estradiol valerate (**CA + E**) reduced ALT blood concentration by 36.02% (*p* < 0.01), both compared with the administration of estradiol valerate (**E**) alone. In the **E** group, Bil-T serum concentration was increased by 56.72% (*p* < 0.0001) compared with **CO** rats, whereas in the **F + E** and **CA + E** groups the same parameter was reduced by 28.57% (*p* < 0.01) and 31.43% (*p* < 0.01), respectively, in comparison with the **E** group of rats. Treatments showed no significant effect on blood GGT level. The combined control (**CC**) group had an increased level of creatinine in the blood (22.28% (*p* < 0.05)) compared with intact control (**IC**) animals. Treatment with flutamide (**F**) caused a reduction in creatinine concentration by 10.42% (*p* < 0.05) compared with the **CV** group, but the treatment of male rats with estradiol valerate (**E**) increased creatinine blood concentration by 40.34% (*p* < 0.0001) compared with **CO** rats. The combined administration of flutamide and estradiol valerate (**F + E**) lowered creatinine level in the blood by 29.15% (*p* < 0.0001) compared with the **CC** group and by 42.33% (*p* < 0.0001) compared with the **E** group. Similarly, serum creatinine level was reduced in the **CA + E** group by 30.56% (*p* < 0.0001) and 43.48% (*p* < 0.0001) compared with the **CC** and **E** groups of rats, respectively. Control vehicle (**CV**) males showed an increase in urea serum concentration by 14.06% (*p* < 0.05) compared with intact control (**IC**) rats, while the combined control (**CC**) group also had an increased concentration of urea by 22.28% (*p* < 0.05) compared with the intact control (**IC**). The treatment of animals with flutamide (**F**) lowered urea levels by 16% (*p* < 0.01) compared with the **CV** group, but the treatment of rats with estradiol valerate (**E**) increased urea levels by 68.14% (*p* < 0.01) compared with the **CO** group. A decrease in urea blood concentration by 28.34% (*p* < 0.05) was observed after cyproterone acetate and estradiol valerate (**CA + E**) administration, compared with estradiol valerate (**E**) alone.

## 4. Discussion

In this research, we elaborate on ‘variations on the theme’ of rat models that simulate therapeutic approaches to prostate cancer and/or sex reassignment. To date, experimental strategies supporting both therapies remain poorly reported and inconsistent, particularly regarding the appropriate dosage of therapeutic compounds in animals relative to human doses. Considering the variety of possible biomedical effects of antiandrogen and estradiol administration on male subjects, and consequently their impact on body composition and metabolic profile (the primary observable set of changes), we conducted evaluations at the level of anthropometric measurements, liver histopathology, and biochemical analysis. As the particular treatments (flutamide, cyproterone acetate, estradiol valerate, as well as their combinations—flutamide + estradiol valerate and cyproterone acetate + estradiol valerate) present an essential pharmaceutical component of their corresponding clinical practice, having an established experimental model in this regard would improve the understanding of each approach, thereby improving the translational potential of future studies. In view of the effect of endogenous testosterone on the increase in lean body mass, as well as the effect of endogenous estradiol on adipose tissue composition and reduced fat accumulation, evaluating the anthropometric parameters presents a rational initial step [29,30]. Macroscopic and histological analysis of the liver was necessary, as it is the site of metabolic clearance and the disposal of xenobiotics. To complete the biochemical assessment of administered compounds, analysis of blood glucose and lipid profiles, liver transaminases, as well as urea and creatinine levels, was conducted. The above three aspects of evaluation/analysis represent a solid foundation for further biomedical research using these exploited models.

Changes in body mass were unidirectional, with no considerable oscillation evident during the experiment. Namely, the control and antiandrogen-administered groups showed a continuous rise in body mass, nearly reaching a plateau by the end of the experiment, which is partly expected since rodents typically attain full growth between 17 and 24 weeks of age [31]. Additionally, this plateau may serve as an indicator that the neuroendocrine system has reached functional maturity. The observed increase in the body mass of intact control rats during the first and second weeks of the experiment (from 350 to 400 g) implies that neurodevelopment, the establishment of hormonal feedback mechanisms, and neuroendocrine integration are still ongoing processes. Although the impairment of androgen receptor (AR) function results in decreased lean body mass and a following increase in WAT and total body mass, the latter may prove more prominent and therefore result in seemingly unaltered growth in the **F** and **CA** groups [29]. This phenomenon, characterized as sarcopenic obesity, is often observed in prostate cancer patients undergoing androgen deprivation therapy [32]. In contrast, estradiol valerate (alone and in combination with both antiandrogens) caused a continuous decline in body mass, which was most prominent after the second week and resumed until the end of the experiment. A decrease in body mass throughout the experiment in groups treated with estradiol valerate (**E**, **F + E**, **CA + E**) is in line with a decrease in WAT mass, given that estradiol regulates WAT through its binding to estrogen receptors (ERs), promoting lipolysis (through the activation of hormone-sensitive lipase) and inhibiting lipogenesis (by decreasing the activity of lipoprotein lipase) [33,34,35]. It was shown that ERαKO (ERα *knockout*) mice had increased WAT mass, as well as hyperplasia and the hypertrophy of white adipocytes, compared with WT mice [30]. After androgen deprivation, which results in increased WAT and decreased lean body mass, and the subsequent application of estradiol to male rats with its mentioned effects in this respect, a further decrease in body mass was to be expected and was realized in our groups receiving combined treatment [29]. Recent data from the literature suggests a positive association between endogenous estradiol and BMI in men, where an increase in endogenous estradiol is followed by an increased BMI, but the current setup resulted in a reduced BMI after estradiol administration in our experiment [36]. Significantly reduced liver and heart mass, as well as reduced testis and prostate mass, could also contribute to the reduction in body mass and consequently BMI after treatment with estradiol valerate, but not as significantly in terms of BMI when it comes to combined treatment with flutamide and cyproterone acetate. Given that androgen deprivation had no effect on heart mass, it would be reasonable to assume that the observed changes are likely a result of estradiol action. Sex hormones can impact the maintenance of normal heart mass, although their exogenous administration did not influence the development of physiological or pathological hypertrophy [37,38]. The treatment of orchidectomized male rats on a high-fat diet with 1 mg/kg/day (for 75 days) estradiol is known to reduce liver mass [39]. It was previously shown that flutamide, at a dose of 5 mg twice daily for 10 days, administered to adult male rats, significantly reduced prostate mass but had no effect on testis mass, while in our study it had no effect on the mass of either organ [40]. Similarly, the administration of cyproterone acetate to male rats reduced testis and prostate mass relative to total body mass, contrary to our observations [41]. Apart from a loss of libido, erectile dysfunction, and decreased penile length, atrophy of the prostate and testis are common clinical outcomes of the antiandrogen treatment of prostate cancer on male reproductive organs [42]. On the other hand, estradiol was shown to greatly reduce testis and ventral prostate mass in adult male rats treated with 17β-estradiol (approximately 14.5 µg/day for 3 months), which is in line with our results [43]. Male-to-female transgender patients receiving estradiol showed decreased diameters of seminiferous tubules, the degeneration of the nucleus and cytoplasm of Leydig cells, and an overall lower number of Leydig cells [44]. Known stimulatory effects of estradiol dipropionate (0.625 mg/kg b.m.) on the hypothalamic–pituitary–adrenal axis of orchidectomized adult male rats resulted in an increased mass of adrenal and a greatly increased mass of pituitary glands, although their impact on total body mass could be considered insignificant [18,20]. A substantial reduction in the specific rate of body mass gain after the administration of estradiol and antiandrogens, especially in the combined treatments, encompasses the aforementioned results. Considering the nature of the parameter, a decrease of more than 100% would imply a loss of mass during the experiment. The lipid profile of the experimental animals is characterized by increased triglycerides in combined treatment groups, increased HDL in the **CA + E** group, and decreased LDL following the administration of estradiol alone and in combination with both antiandrogens. Previous studies reported similar changes in serum triglyceride and HDL concentrations, but they also observed increased LDL in orchidectomized male rats treated with estradiol dipropionate (0.6 mg/kg b.m.), the latter being in contradiction with our finding [21]. However, men undergoing estrogen therapy for prostate cancer showed the same pattern of change in terms of triglyceride, HDL, and LDL levels as observed in our study [45]. Dyslipidemia is one of key factors in the development of metabolic syndrome and consequently increased cardiovascular morbidity in subjects receiving either androgen deprivation therapy or gender-affirming estrogen therapy [32,46].

The results of our previous study showed increased absolute and relative liver mass after the experimental estrogenization of orchidectomized male rats, while in the current setup, absolute liver mass was reduced in the estradiol valerate and combined treatment groups [18]. However, relative liver mass was increased in this respect which could be a result of a more prominent decrease in body mass and reduced WAT after androgen deprivation and estrogenization. Relative organ mass should be considered a more accurate representation of chronic treatment’s effect on the organ of interest, as it reflects the organ’s contribution to total body mass. In light of the known hepatotoxic implications the antiandrogen compounds have, the administered doses caused no evident superficial changes to hepatic tissue or overall liver morphology. At the level of liver histology, changes were not pronounced while morphometry revealed a significant elevation in the volume density of binuclear hepatocytes, as well as connective tissue after combined estrogen–antiandrogen treatment. An increased percentage of binuclear hepatocytes under heightened estradiol and antiandrogen exposure indicates an adaptive hepatocellular response, reflecting cellular attempts to maintain liver function under sustained pharmacological stress. Considering the liver is heavily engaged in drug detoxification, a higher percentage of binuclear hepatocytes is to be expected in our study, as a polyploid genome in liver cells may act as a protective mechanism against drug-induced mutations and the expression of harmful genes [47]. Additionally, the combined treatment appears to induce low-grade hepatocellular stress, characterized by increased ROS generation, inflammatory signaling, and lipid accumulation. These changes are known to activate hepatic stellate cells and initiate a fibrogenic program [48]. Accordingly, we observed a modest but significant increase in connective tissue volume density, indicative of extracellular matrix remodeling, with Masson’s staining revealing blue collagen fibers predominantly around the central vein and the portal triad. From the perspective of PAS staining, the glycogenic profile appears to be uniform across all experimental groups and glycogen depots seem well preserved, although it has previously been reported that castration inhibits glycogen synthesis, while estrogen administration to castrated rats showed no effect accordingly [49]. The preservation of the glycogen depots in our experimental context could be a result of *ad libitum* access to food and its considerable consumption.

Serum liver enzyme levels, commonly used as markers of hepatocellular injury, were not altered following cyproterone acetate and flutamide administration alone, whereas antiandrogens combined with estradiol valerate resulted in reduced ALT and AST concentrations. Estradiol can modulate the metabolic status of hepatocytes, including the expression, release, and systemic clearance of aminotransferases, as well as the transcription of enzymes involved in aminotransferase metabolism. Antiandrogens, on the other hand, may reduce aminotransferase induction through androgen receptor-dependent pathways [50]. Together, these processes can result in a reduced basal secretion of ALT and AST into circulation. In line with the observed histological changes following the combined treatments (**F + E** and **CA + E**), the decrease in ALT/AST values therefore reflects a predominantly metabolic and adaptive hepatic response rather than a cytotoxic reaction accompanied by hepatocellular necrosis. The increased proportion of binucleated hepatocytes further supports enhanced metabolic and regenerative activity, helping maintain cell membrane integrity and preventing the leakage of intracellular enzymes [51]. Our results also indicate that low-grade fibrosis is not directly associated with membrane damage or enzyme release. The literature consistently notes that reduced or within-range ALT/AST values lack clinical significance and do not necessarily correlate with hepatic injury [52]. On the other hand, patients undergoing total androgen blockade (flutamide + LHRH analog) for prostate cancer showed greatly increased ALT, AST, and bilirubin levels [53,54]. Similarly, cyproterone acetate at variable doses increased AST, ALP, and total bilirubin in prostate cancer patients during therapy, while in our study only serum ALP levels were greatly increased [55]. Estradiol at a dose of 0.5 mg/kg b.m. daily (14 days) reduced collagen accumulation during dimethylnitrosamine-induced hepatic fibrosis in male rats, and it can also reduce AST and ALT serum concentrations in diabetic male rats, which could explain the significant reduction in liver enzymes even with antiandrogen administration in combination with estradiol valerate [56,57]. Previous reports showed a similar pattern of decreased blood ALT and AST concentrations in trans-females after estradiol administration [58]. However, bearing in mind the relatively short half-life of liver enzymes in circulation and the fact that some are also found in other tissues (i.e., AST is also found in the heart and skeletal muscle), considering them as precise indicators of liver damage may not be completely accurate.

Treatment with flutamide and estradiol has been shown to decrease absolute kidney mass, whereas estradiol alone, as well as combined with cyproterone acetate, caused a significant increase in relative kidney mass. This increase is most likely due to decreased total body mass relative to the kidney, but it may also result from aldosterone-mediated glomerular cell hypertrophy and renal fibrosis, leading to tissue rearrangement and an increase in mass, which was seen with rats infused with aldosterone [59]. Estradiol is known to increase aldosterone production in the adrenal glands via its central effect on ACTH secretion from the pituitary gland [18]. Elevated levels of urea in the control vehicle and combined control groups, as well as elevated creatinine in the combined control group, could be caused by increased stress from treatment administration and rat handling, particularly in combined control animals that received two injections daily throughout the experiment. Flutamide treatment caused a reduction in serum creatinine level; however, a previous study reported no change in blood creatinine concentration after the treatment of orchidectomized rats with flutamide (30 mg/kg b.m. daily for 16 days) [60]. Estradiol is usually considered to have a protective role in the kidneys, but after estradiol valerate treatment, blood urea and creatinine concentrations were increased, whereas in combination with antiandrogen they were decreased [61]. This could suggest a stronger effect of testosterone over estradiol on renal function in males, as males have shown faster progression of renal injury compared with females in general [62]. Although urea and creatinine are considered common indicators of kidney function, they are influenced by many external factors and provide only a crude evaluation of the current state of the kidney [63].

## 5. Conclusions

Numerous effects of androgen deprivation and estrogenization are summarized in Table 8. Given that gonadal steroids strongly influence body composition, the most pronounced changes in body mass, WAT mass, organ mass, and lipid profile were observed in groups treated with estradiol valerate alone or in combination with antiandrogens. Furthermore, the administration of estradiol and antiandrogens together caused significant changes in liver mass, likely due to increased metabolic strain and known hepatotoxic implications, particularly of flutamide and cyproterone acetate, on the liver. Under sustained pharmacological stress, changes in liver histology, including an increase in the volume density of binuclear hepatocytes and connective tissue, as well as alterations in biochemical parameters, indicate an adaptive hepatocellular response. In the context of the kidneys, chronic estradiol exposure led to elevated serum urea and creatinine levels, while combined treatments had a less detrimental effect on these kidney parameters and male metabolism in general. The observed alterations may predispose the male organism to adverse long-term outcomes, and more extensive toxicological evaluation, both immediate and prolonged, is required. Bearing in mind the importance of our experimental treatments and their relevance to clinical practice, a detailed report of their effects on basic morpho-physiological parameters is highly beneficial for future studies on the impact of these treatments on certain organs or organ systems of interest.

## Figures and Tables

**Table 1:** Brief comparison of chemical and surgical castration approaches in animal models.

	Intervention	Chemical Castration	Surgical Castration
Aspect			
**Similarity to human therapies**		Closely mimics clinical androgen deprivation therapy or sex reassignment hormone therapy	Mimics clinical orchidectomy-only scenarios
**Reversibility**		**Reversible**—testicular function and fertility can recover after cessation of treatment	**Irreversible**—permanent loss of fertility
**Experimental flexibility**		Allows dose appropriation and combining of agents; therapy can be stopped for recovery observation	One fixed endpoint; modification is not possible after castration
**Endocrine control**		Adjustable, modifiable—estradiol can be added to achieve specific hormonal states	Produces uniform, very low levels of endogenous testosterone without the option of fine control

**Table 2:** Equations used for calculating body mass index (BMI, g/cm^2^), Lee’s index (g/cm), adiposity index (n.a.), and specific rate of body mass gain (g/kg) [14].

Parameter	Unit	Equation
Body mass index (BMI)	g/cm^2^	$\frac{Body\;mass\;(g)}{{Length}^{2}\;({cm}^{2})}$
Lee’s index	g/cm	$\frac{\sqrt[{3}]{Body\;mass\;(g)}}{Nose-to-anus\;length\;(cm)}$
Adiposity index	n.a.	$\frac{gWAT\;mass+iWAT\;mass+pWAT\;mass}{Body\;mass}\times 100$
Specific rate of body mass gain	g/kg	$\frac{Final\;body\;mass\;\left(g\right)-initial\;body\;mass\;(g)}{Initial\;body\;mass\;(kg)}$

**Table 3:** Final body mass, nose-to-anus (full body) length, and absolute and relative white adipose tissue WAT mass (gWAT, iWAT, pWAT, and totalWAT) in the control and experimental groups.

	Parameter	Final Body Mass (g)	Nose-to-Anus (Full Body) Length (cm)	Absolute gWAT Mass (g)	Absolute iWAT Mass (g)	Absolute pWAT Mass (g)	Absolute totalWAT Mass (g)	Relative gWAT Mass (g/g b.m.)	Relative iWAT Mass (g/g b.m.)	Relative pWAT Mass (g/g b.m.)	Relative totalWAT Mass (g/g b.m.)
Group											
**Intact control (IC)**		430.2 ± 51.3	25.8 ± 1.3	2.78 ± 0.28	1.94 ± 1.17	8.02 ± 1.65	12.60 ± 1.84	0.66 ± 0.15	0.45 ± 0.27	1.90 ± 0.53	3.01 ± 0.70
**Control vehicle (CV)**		420.2 ± 50.5	25.8 ± 0.8	2.13 ± 0.52 * (−23.49% vs. IC) ↓	1.57 ± 0.53	10.03 ± 3.30	13.72 ± 3.15	0.50 ± 0.09	0.37 ± 0.11	2.42 ± 0.85	3.29 ± 0.82
**Cyproterone acetate (CA)**		427.2 ± 20.1	26.0 ± 1.0	3.23 ± 0.40 *** (+51.86% vs. CV)	0.90 ± 0.25	8.63 ± 2.09	12.76 ± 2.30	0.75 ± 0.08 ** (+44.6% vs. CV)	0.21 ± 0.05	2.01 ± 0.43	2.97 ± 0.44
**Flutamide (F)**		415.2 ± 26.1	25.8 ± 1.1	2.48 ± 0.26 ↑	0.73 ± 0.41	6.63 ± 1.43	9.84 ± 2.03	0.60 ± 0.03 ↑	0.17 ± 0.09	1.60 ± 0.27	2.38 ± 0.35
**Control sesame oil (CO)**		433.0 ± 17.4	26.0 ± 0.0	2.50 ± 0.38	1.29 ± 0.30	11.70 ± 3.73	15.49 ± 3.82	0.58 ± 0.10	0.30 ± 0.07	2.70 ± 0.82	3.57 ± 0.83
**Estradiol valerate (E)**		309.0 ± 33.4 *** (−28.64% vs. CO) ↓	23.0 ± 1.2 ** (−11.54% vs. CO)	1.23 ± 0.43 *** (−50.84% vs. CO) ↓	0.81 ± 0.25	7.48 ± 1.95 * (−36.01% vs. CO) ↓	9.52 ± 2.47 * (−38.54% vs. CO) ↓	0.41 ± 0.16	0.27 ± 0.10	2.43 ± 0.67	3.10 ± 0.90
**Combined control (CC)**		399.0 ± 35.9	25.0 ± 1.0	2.24 ± 0.41 * (−19.46% vs. IC) ↓	1.61 ± 1.00	6.17 ± 2.60	10.02 ± 3.80	0.57 ± 0.12	1.53 ± 0.62	1.89 ± 0.46	2.50 ± 0.91
**Flutamide + Estradiol valerate** **(F + E)**		276.0 ± 9.0 **** (−30.83% vs. CC) ↓	21.8 ± 0.8 *** (−12.8% vs. CC) ↓	1.51 ± 0.20 ** (−32.51% vs. CC) ↓	0.59 ± 0.16	7.44 ± 1.32	9.54 ± 1.22	0.55 ± 0.06	2.72 ± 0.47 * (+77.42% vs. CC) ↑	2.64 ± 0.15	3.50 ± 0.42
**Cyproterone acetate + Estradiol valerate (CA + E)**		273.2 ±14.0 **** (−31.53% vs. CC) ↓	21.6 ± 0.5 *** (−13.6% vs. CC) ↓	1.34 ± 0.13 *** (−40.37% vs. CC) ↓	0.75 ± 0.20	7.06 ± 2.90	9.15 ± 3.02	0.49 ± 0.04	2.56 ± 0.10	2.29 ± 0.33	3.32 ± 1.00

All values are presented as mean ± SD, n = 5 animals *per* group. * *p* < 0.05, ** *p* < 0.01, *** *p* < 0.001, **** *p* < 0.0001. Red arrow indicates increase of parameters, while blue arrow indicates decrease of parameters.

**Table 4:** Absolute organ mass in the control and experimental groups.

	Parameter	Absolute Organ Mass						
Group		Pituitary (mg)	Left Adrenal Gland (mg)	Liver (g)	Left Kidney (mg)	Prostate (mg)	Heart (mg)	Testis (random; mg)
**Intact control (IC)**		12.20 ± 0.45	30.80 ± 3.70	13.07 ± 0.93	1.28 ± 0.18	1.96 ± 0.19	1.29 ± 0.17	2.05 ± 0.28
**Control vehicle (CV)**		11.40 ± 1.95	28.40 ± 4.72	14.08 ± 1.59	1.27 ± 0.20	1.67 ± 0.45	1.40 ± 0.14	2.04 ± 0.21
**Cyproterone acetate (CA)**		13.60 ± 0.90	27.80 ± 1.48	14.94 ± 0.69	1.31 ± 0.07	1.56 ± 0.32	1.34 ± 0.06	2.06 ± 0.06
**Flutamide (F)**		12.25 ± 1.71	33.75 ± 4.35	13.78 ± 0.67	1.25 ± 0.06	1.81 ± 0.16	1.33 ± 0.05	2.13 ± 0.15
**Control sesame oil (CO)**		13.20 ± 4.60	30.60 ± 1.52	14.54 ± 0.79 * (+11.25% vs. IC) ↑	1.26 ± 0.07	1.88 ± 0.18	1.29 ± 0.06	2.18 ± 0.10
**Estradiol valerate (E)**		47.60 ± 11.10 **** (+3.61× vs. CO) ↑	36.00 ± 4.30 * (+17.65% vs. CO) ↑	9.65 ± 0.83 **** (−33.61% vs. CO) ↓	1.10 ± 0.22	0.17 ± 0.03 **** (−91.17% vs. CO) ↓	0.98 ± 0.16 ** (−23.72% vs. CO) ↓	0.35 ± 0.03 **** (−83.74% vs. CO) ↓
**Combined control (CC)**		11.00 ± 1.58	32.60 ± 3.78	13.34 ± 2.10	1.30 ± 0.26	1.84 ± 0.12	1.32 ± 0.21	1.75 ± 0.16 * (−15.02% vs. IC)
**Flutamide + Estradiol valerate (F + E)**		39.25 ± 11.53 **** (+3.57× vs. CC) ↑	27.75 ± 4.57	10.10 ± 0.92 ** (−24.29% vs. CC) ↓	0.97 ± 0.10 * (−25.41% vs. CC) ↓	0.17 ± 0.01 **** (−90.5% vs. CC) ↓	0.82 ± 0.05 *** (−38.01% vs. CC) ↓	0.29 ± 0.01 **** (−83.58% vs. CC) * (−19.2% vs. E) ↓
**Cyproterone acetate + Estradiol valerate (CA + E)**		32.00 ± 7.71 *** (+2.91× vs. CC) ↑	31.40 ± 5.60	10.72 ± 0.95 * (−19.64% vs. CC) ↓	1.03 ± 0.13	0.16 ± 0.02 **** (−91.3% vs. CC) ↓	0.85 ± 0.09 *** (−34.82% vs. CC) ↓	0.34 ± 0.05 **** (−80.7% vs. CC) ↓

All values are presented as mean ± SD, n = 5 animals *per* group. * *p* < 0.05, ** *p* < 0.01, *** *p* < 0.001, **** *p* < 0.0001. Red arrow indicates increase of parameters, while blue arrow indicates decrease of parameters.

**Table 5:** Relative organ mass in the control and experimental groups.

	Parameter	Relative Organ Mass						
Group		Pituitary (mg/g b.m.)	Left Adrenal Gland (mg/g b.m.)	Liver (g/g b.m.)	Left Kidney (mg/g b.m.)	Prostate (mg/g b.m.)	Heart (mg/g b.m.)	Testis (random; mg/g b.m.)
**Intact control (IC)**		2.87 ± 0.36	7.19 ± 0.68	3.06 ± 0.22	0.30 ± 0.01	0.46 ± 0.08	0.30 ± 0.01	0.48 ± 0.04
**Control vehicle (CV)**		2.72 ± 0.31	6.75 ± 0.54	3.35 ± 0.05 * (+17.45% vs. IC) ↑	0.30 ± 0.01	0.39 ± 0.06	0.34 ± 0.04	0.49 ± 0.05
**Cyproterone acetate (CA)**		3.18 ± 0.16	6.52 ± 0.38	3.50 ± 0.10	0.31 ± 0.01	0.37 ± 0.08	0.31 ± 0.01	0.49 ± 0.03
**Flutamide (F)**		2.97 ± 0.36	8.17 ± 0.53 ** (+21.15% vs. CV) ↑	3.35 ± 0.17	0.31 ± 0.01	0.44 ± 0.05	0.33 ± 0.03	0.52 ± 0.06
**Control sesame oil (CO)**		3.06 ± 1.09	7.08 ± 0.54	3.36 ± 0.11	0.29 ± 0.02	0.44 ± 0.05	0.30 ± 0.02	0.50 ± 0.04
**Estradiol valerate (E)**		15.78 ± 4.71 **** (+5.16× vs. CO) ↑	11.67 ± 0.90 **** (+64.78 vs. CO) ↑	3.15 ± 0.43	0.35 ± 0.03 ** (+20.37% vs. CO) ↑	0.05 ± 0.02 **** (−87.42% vs. CO) ↓	0.32 ± 0.03	0.12 ± 0.01 **** (−77.14% vs. CO) ↓
**Combined control (CC)**		2.76 ± 0.29	8.17 ± 0.54	3.33 ± 0.26	0.32 ± 0.04	0.46 ± 0.05	0.33 ± 0.04	0.44 ± 0.05
**Flutamide + Estradiol valerate (F + E)**		14.41 ± 4.40 **** (+5.23× vs. CC) ↑	10.14 ± 1.62	3.70 ± 0.23 * (+17% vs. E)	0.35 ± 0.03	0.06 ± 0.002 **** (−86.35% vs. CC) ↓	0.30 ± 0.01	0.10 ± 0.006 **** (−76.37% vs. CC) ↓
**Cyproterone acetate + Estradiol valerate (CA + E)**		11.74 ± 3.00 *** (+4.26× vs. CC) ↑	11.47 ± 1.81 ** (+40.46% vs. CC) ↑	3.92 ± 0.23 ** (+17.75% vs. CC) ** (+24.33% vs. E) ↑	0.38 ± 0.04 * (+15.96% vs. CC) ↑	0.06 ± 0.01 **** (−87.34% vs. CC) ↓	0.31 ± 0.03	0.12 ± 0.02 **** (−72.04% vs. CC) ↓

All values are presented as mean ± SD, n = 5 animals *per* group. * *p* < 0.05, ** *p* < 0.01, *** *p* < 0.001, **** *p* < 0.0001. Red arrow indicates increase of parameters, while blue arrow indicates decrease of parameters.

**Table 6:** Serum concentrations of glucose, triglycerides, HDL, and LDL in the following: **IC**—intact control, **CV**—control vehicle, **CA**—cyproterone acetate, **F**—flutamide, **CO**—control sesame oil, **E**—estradiol valerate, **CC**—combined control, **F + E**—flutamide + estradiol valerate, **CA + E**—cyproterone acetate + estradiol valerate groups of rats.

	Parameter	Glucose (mmol/L)	Triglycerides (mmol/L)	HDL (mmol/L)	LDL (mmol/L)
Group					
**Intact control (IC)**		6.46 ± 0.35	1.28 ± 0.17	0.72 ± 0.06	0.32 ± 0.08
**Control vehicle (CV)**		7.30 ± 0.84	1.87 ± 0.49 * (+55.06% vs. IC) ↑	0.77 ± 0.03	0.45 ± 0.10
**Cyproterone acetate (CA)**		6.72 ± 0.97	1.67 ± 0.31	0.90 ± 0.17	0.36 ± 0.05
**Flutamide (F)**		7.45 ± 0.73	1.47 ± 0.29	0.70 ± 0.56	0.38 ± 0.10
**Control sesame oil (CO)**		6.60 ± 0.64	1.82 ± 0.50	0.77 ± 0.06	0.34 ± 0.05
**Estradiol valerate (E)**		5.92 ± 0.37	1.70 ± 0.21	1.00 ± 0.29	0.17 ± 0.05 ** (−47.5% vs. CO) ↓
**Combined control (CC)**		6.32 ± 0.72	1.10 ± 0.24	0.67 ± 0.11	0.48 ± 0.08 ** (+50% vs. IC) ↑
**Flutamide + Estradiol valerate (F + E)**		5.90 ± 0.59	2.16 ± 0.59 ** (+95.65% vs. CC) ↑	0.79 ± 0.16	0.10 ± 0.00 **** (−79.17% vs. CC) ** (−40.48% vs. E) ↓
**Cyproterone acetate + Estradiol valerate (CA + E)**		6.30 ± 0.62	2.30 ± 0.45 *** (+107.97% vs. CC)	0.88 ± 0.11 ** (+30.65% vs. CC) ↑	0.10 ± 0.00 **** (−79.17% vs. CC) ** (−40.48% vs. E) ↓

All values are presented as mean ± SD, n = 5 animals *per* group. * *p* < 0.05, ** *p* < 0.01, *** *p* < 0.001, **** *p* < 0.0001. Red arrow indicates increase of parameters, while blue arrow indicates decrease of parameters.

**Table 7:** Serum concentrations of ALP, AST, ALT, Bil-T, GGT, creatinine, and urea in the following: **IC**—intact control, **CV**—control vehicle, **CA**—cyproterone acetate, **F**—flutamide, **CO**—control sesame oil, **E**—estradiol valerate, **CC**—combined control, **F + E**—flutamide + estradiol valerate, **CA + E**—cyproterone acetate + estradiol valerate groups of rats.

	Parameter	ALP (U/L)	AST (U/L)	ALT (U/L)	Bil-T (µmol/L)	GGT (U/L)	Creatinine (µmol/L)	Urea (mmol/L)
Group								
**Intact control (IC)**		307.60 ± 55.42	409.70 ± 59.77	96.34 ± 8.16	1.40 ± 0.21	2.46 ± 1.21	62.00 ± 2.55	7.54 ± 0.31
**Control vehicle (CV)**		264.90 ± 23.86	409.20 ± 23.68	92.50 ± 9.32	1.63 ± 0.21	4.13 ± 2.25	64.75 ± 4.99	8.60 ± 0.16 * (+14.06% Vs. IC) **↑**
**Cyproterone acetate (CA)**		511.60 ± 89.89 *** (+93.13% Vs. CV) **↑**	417.40 ± 74.64	113.50 ± 17.32	1.32 ± 0.18	3.18 ± 0.79	59.00 ± 3.94	8.04 ± 0.84
**Flutamide (F)**		315.50 ± 53.98	427.90 ± 41.02	82.05 ± 41.28	1.35 ± 0.17	3.15 ± 0.54	58.00 ± 2.45 * (−10.42% Vs. CV) **↓**	7.23 ± 0.22 ** (−16% Vs. CV) **↓**
**Control sesame oil (CO)**		331.20 ± 39.83	451.10 ± 108.40	107.00 ± 12.59	1.34 ± 0.11	3.74 ± 1.06	59.00 ± 3.39	7.22 ± 0.73
**Estradiol valerate (E)**		152.30 ± 41.83 *** (−54.02% Vs. CO) **↓**	501.20 ± 97.31	142.60 ± 38.31	2.10 ± 0.16 **** (+56.72% Vs. CO) **↑**	3.98 ± 1.40	82.80 ± 4.32 **** (+40.34% Vs. CO) **↑**	12.14 ± 3.10 ** (+68.14% Vs. CO) **↑**
**Combined control (CC)**		301.10 ± 44.39	410.00 ± 70.09	96.94 ± 13.51	1.76 ± 0.42	2.86 ± 1.05	67.40 ± 4.22 * (+22.28% Vs. IC) **↑**	9.22 ± 0.80 * (+22.28% Vs. IC) **↑**
**Flutamide + Estradiol valerate (F + E)**		218.50 ± 33.45	353.30 ± 72.68 * (−29.53% Vs. E) **↓**	95.50 ± 2.06 * (−33.03% Vs. E) **↓**	1.50 ± 0.22 ** (−28.57% Vs. E) **↓**	4.60 ± 1.71	47.75 ± 2.99 **** (−29.15% vs. CC) **** (−42.33% Vs. E) **↓**	10.18 ± 1.14
**Cyproterone acetate + Estradiol valerate (CA + E)**		182.80 ± 57.85 ** (−39.29% Vs. CC) **↓**	295.80 ± 46.04 * (−27.85% vs. CC) ** (−40.98% Vs. E) **↓**	91.24 ± 3.42 ** (−36.02% Vs. E) **↓**	1.44 ± 0.26 ** (−31.43% Vs. E) **↓**	4.46 ± 0.43	46.80 ± 2.77 **** (−30.56% vs. CC) **** (−43.48% Vs. E) **↓**	8.70 ± 0.88 * (−28.34% Vs. E) **↓**

All values are presented as mean ± SD, n = 5 animals *per* group. * *p* < 0.05, ** *p* < 0.01, *** *p* < 0.001, **** *p* < 0.0001. Red arrow indicates increase of parameters, while blue arrow indicates decrease of parameters.

**Table 8:** Summary of the change in evaluated parameters in treated groups compared to their corresponding controls. Every experimental group is displayed in a different color, while color gradient correlates with significance of parameter change (high intensity—**** *p* < 0.0001, medium intensity—*** *p* < 0.001, low intensity—** *p* < 0.01 and * *p* < 0.05).

	Group	CA	F	E	F + E	CA + E
Analyzed Parameter						
Final body mass		∕	∕	↓	↓	↓
Relative organ weight	Pituitary	∕	∕	↑	↑	↑
	Left adrenal gland	∕	↑	↑	∕	↑
	Liver	∕	∕	∕	∕	↑
	Left kidney	∕	∕	↑	∕	↑
	Prostate	∕	∕	↓	↓	↓
	Heart	∕	∕	∕	∕	∕
	Testis	∕	∕	↓	↓	↓
Histological parameters	Binuclear hepatocytes volume density	∕	∕	∕	↑	↑
	Liver PAS staining intensity	∕	∕	∕	∕	∕
	Liver collagen volume density	∕	∕	∕	↑	↑
Serum biochemical parameters	Glucose	∕	∕	∕	∕	∕
	Triglycerides	∕	∕	∕	↑	↑
	HDL	∕	∕	∕	∕	↑
	LDL	∕	∕	↓	↓	↓
	Hepatic enzymes	↑	∕	↓	↓	↓
	Markers of hepatobiliary function	∕	∕	↑	↓	↓
	Markers of kidney function	∕	↓	↑	↓	↓

## Data Availability

Dataset available on request from the authors.

## Supplementary Materials

The following supporting information can be downloaded at: https://www.mdpi.com/article/10.3390/medicina62010008/s1, Supplementary S1: Questionary regarding animal health (Excel tables), Supplementary S2: Unedited photographs of liver macroscope and liver histological micrographs, Supplementary S3: JPEG files of detailed protocols for each biochemical analysis.

## Abbreviations

The following abbreviations are used in this manuscript:

AR	Androgen receptor
LHRH	Luteinizing hormone releasing hormone
WAT	White adipose tissue
BMI	Body mass index
HDLs	High-density lipoproteins
LDLs	Low-density lipoproteins
ALP	Alkaline phosphatase
ALT	Alanine aminotransferase
AST	Aspartate aminotransferase
Bil-T	Bilirubin
GGT	Gamma-glutamil transferase
ER	Estrogen receptor
ERαKO	Estrogen receptor α *knockout*
WT	Wild type
ACTH	Adrenocorticotropic hormone
