# Supplementary figures and images for: Male Rat Model of Chemical Androgen Deprivation and Estrogenization from the Perspective of Anthropometric, Histological, and Biochemical Parameters
Source: Medicina (Kaunas). 2025 Dec 19;62(1):8. doi: 10.3390/medicina62010008 (PMC12842721; doi:10.3390/medicina62010008)

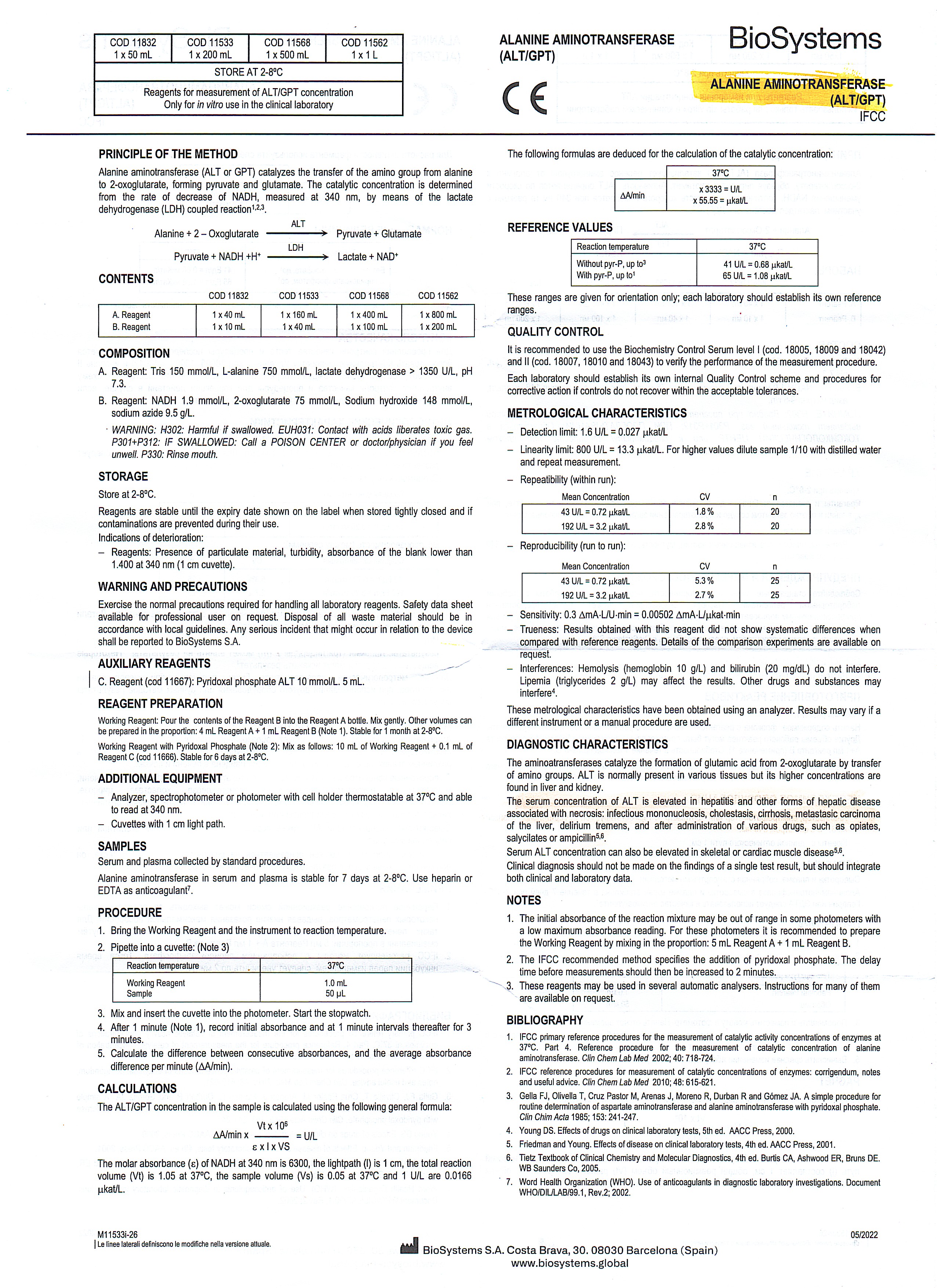

Supplement: Supplementary file 1 [file medicina-62-00008-s001.zip › Supplementary S3/Protocol - alanine aminotransferase (ALT).jpg]

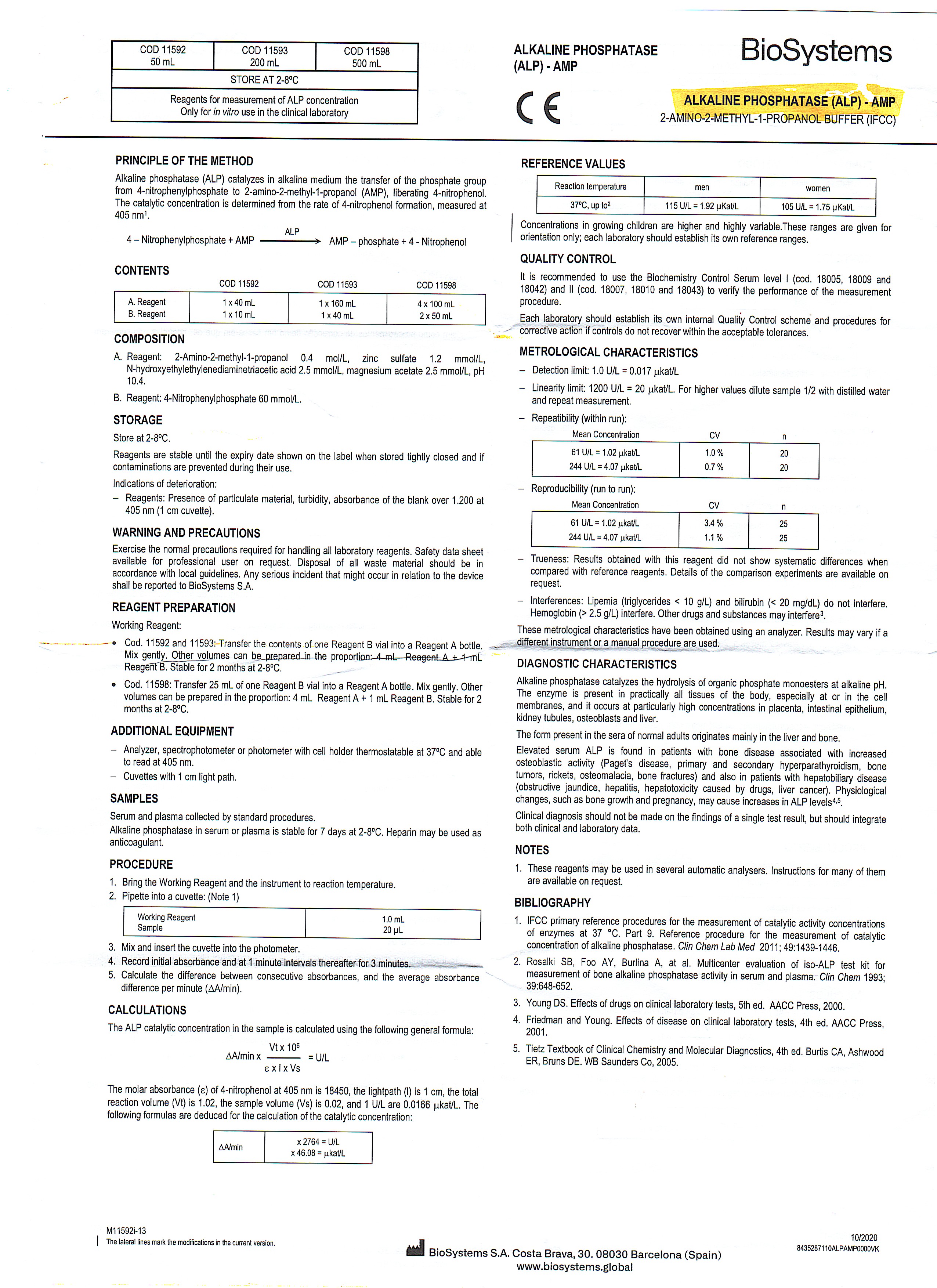

Supplement: Supplementary file 1 [file medicina-62-00008-s001.zip › Supplementary S3/Protocol - alkaline phosphatase (ALP).jpg]

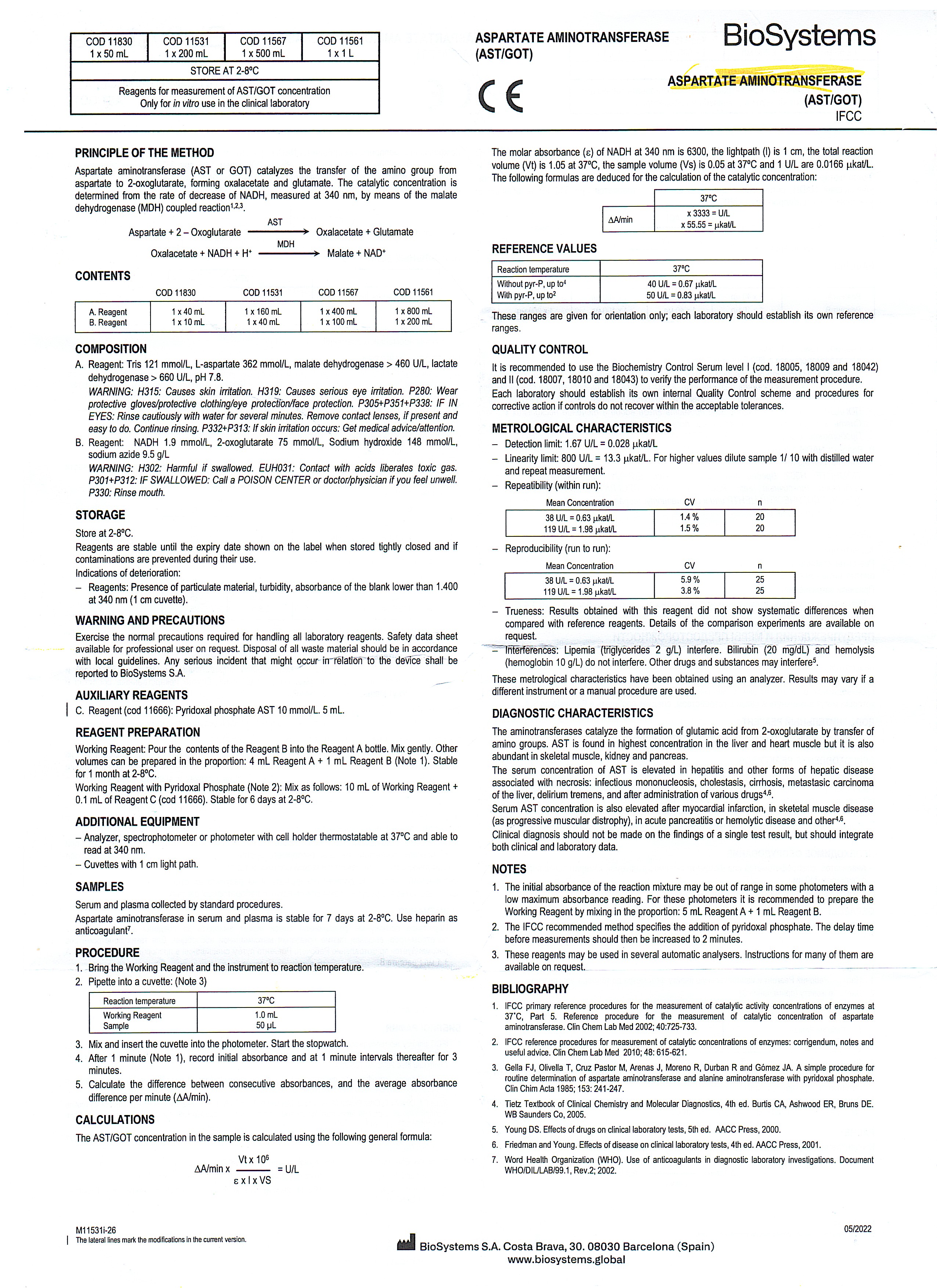

Supplement: Supplementary file 1 [file medicina-62-00008-s001.zip › Supplementary S3/Protocol - aspartate aminotransferase (AST).jpg]

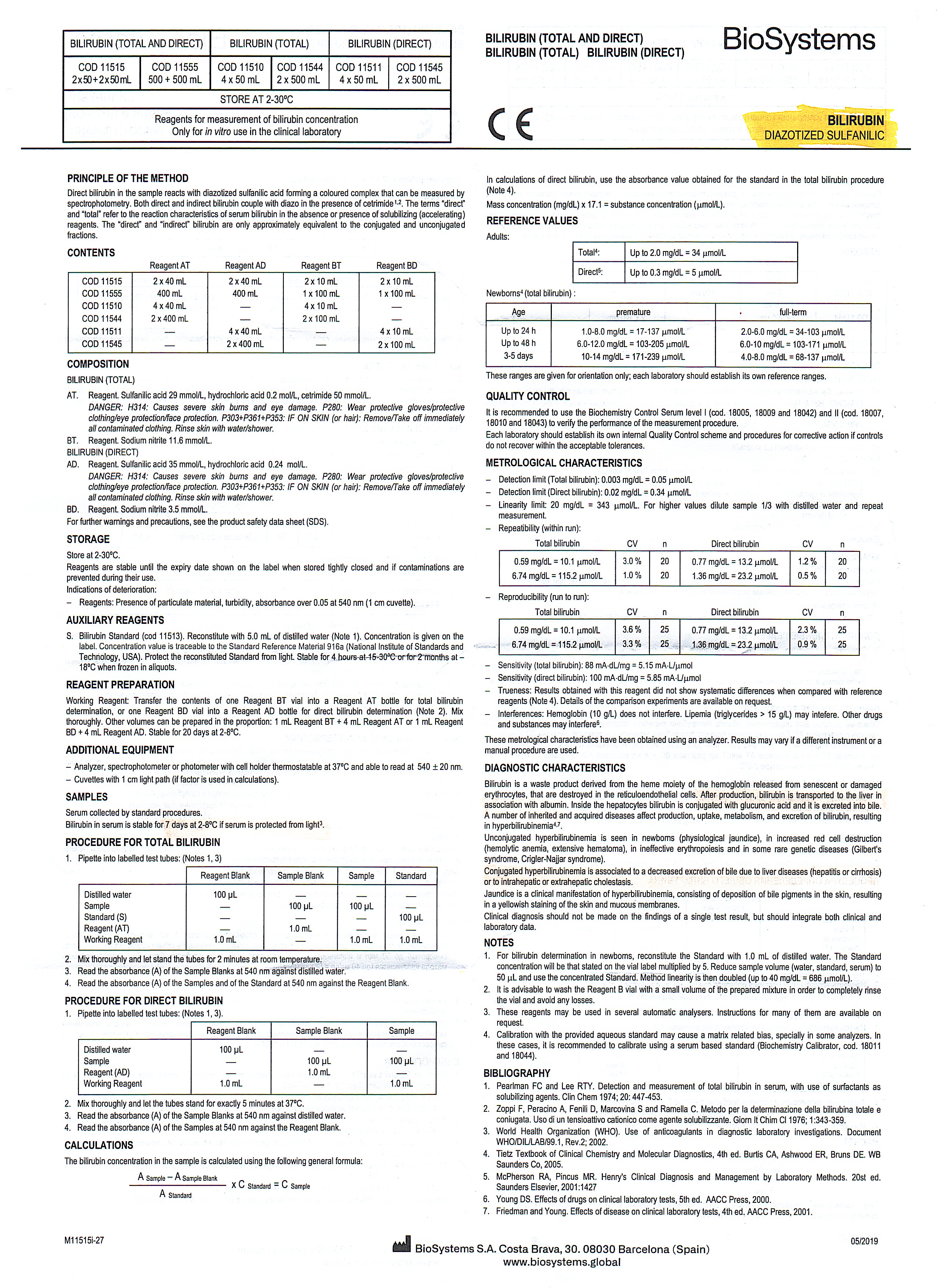

Supplement: Supplementary file 1 [file medicina-62-00008-s001.zip › Supplementary S3/Protocol - bilirubin (Bil-T).jpg]

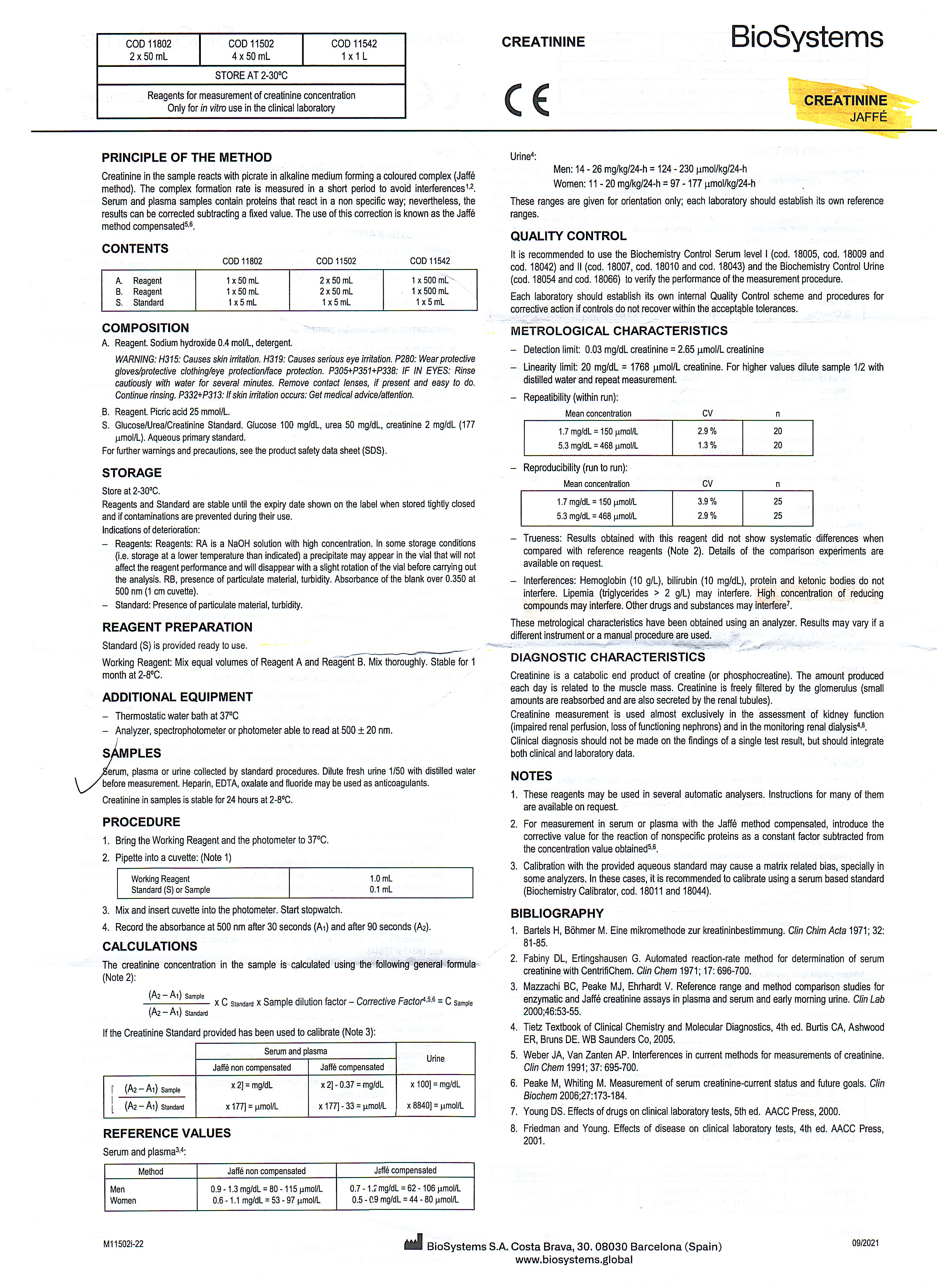

Supplement: Supplementary file 1 [file medicina-62-00008-s001.zip › Supplementary S3/Protocol - creatinine.jpg]

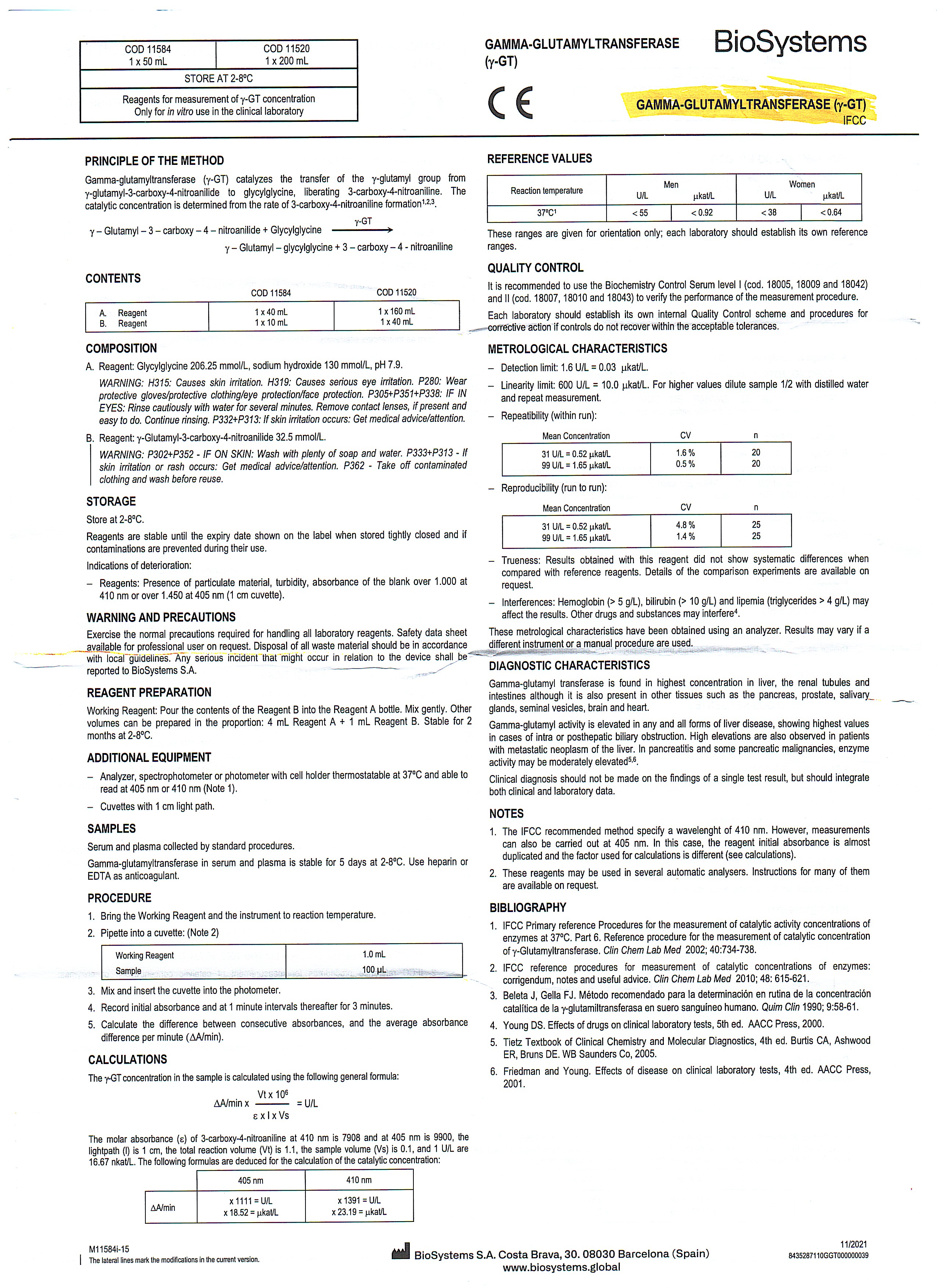

Supplement: Supplementary file 1 [file medicina-62-00008-s001.zip › Supplementary S3/Protocol - gamma-glutamil transferase (GGT).jpg]

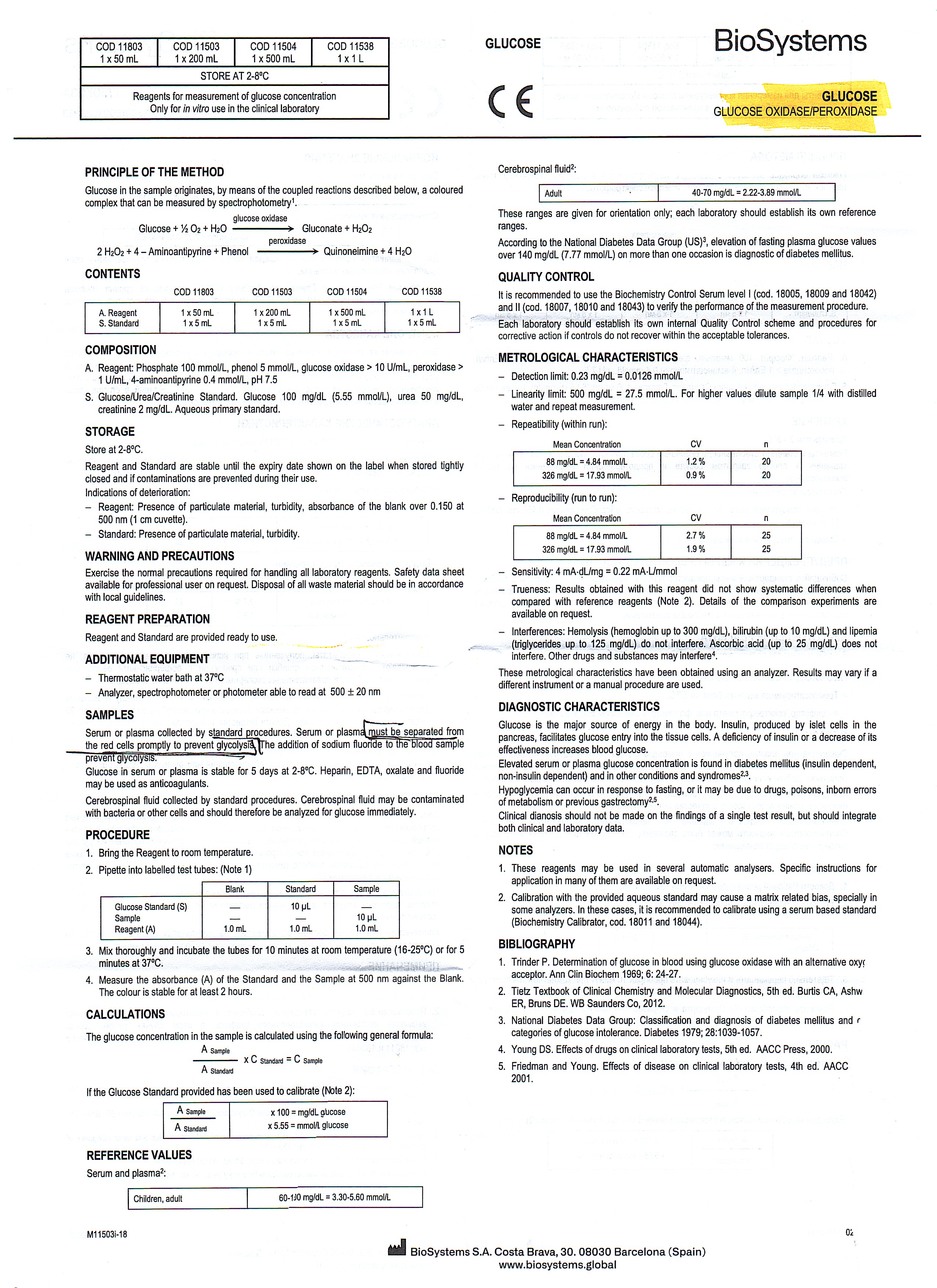

Supplement: Supplementary file 1 [file medicina-62-00008-s001.zip › Supplementary S3/Protocol - glucose.jpg]

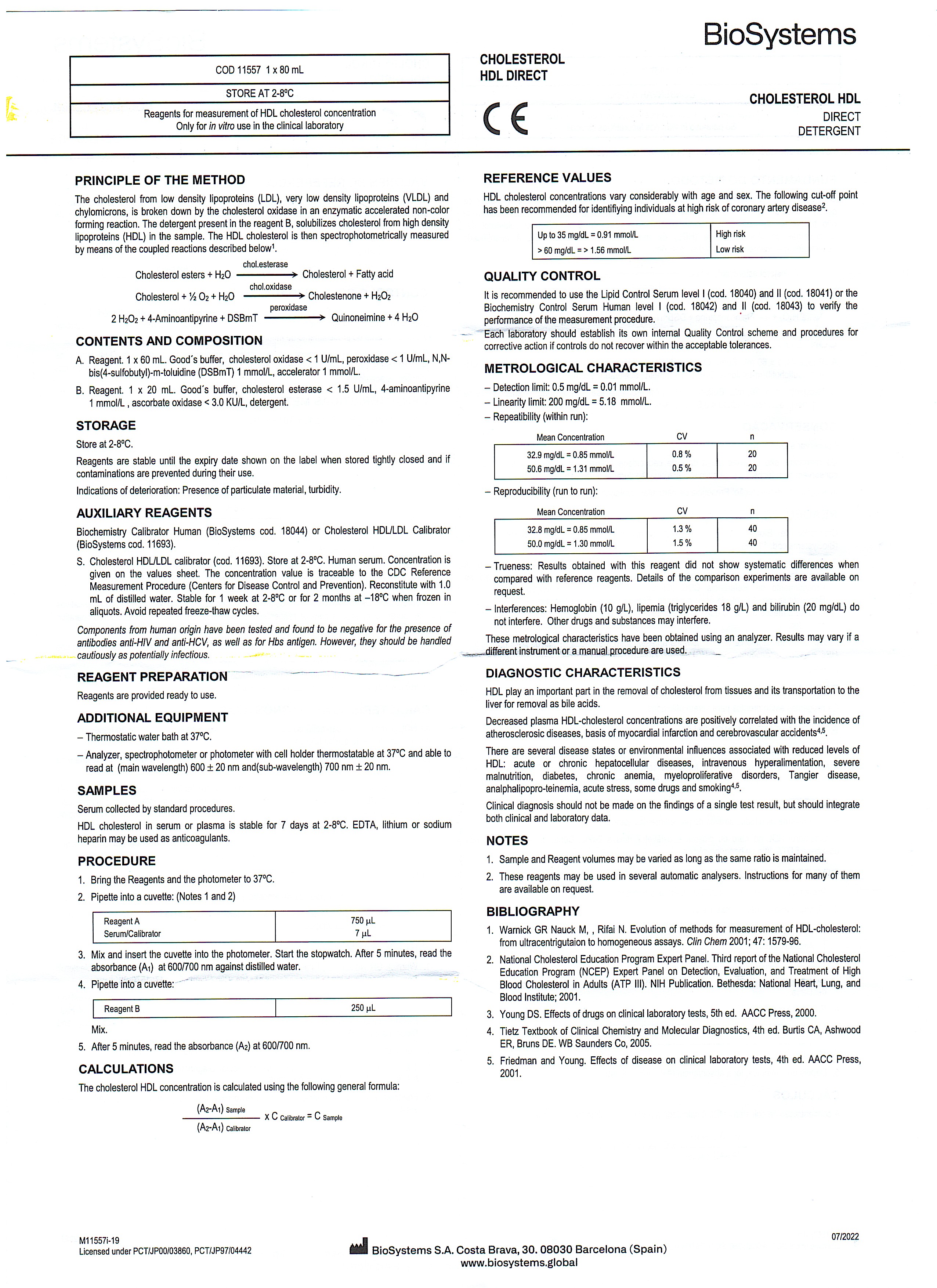

Supplement: Supplementary file 1 [file medicina-62-00008-s001.zip › Supplementary S3/Protocol - high-density lipoproteins (HDL).jpg]

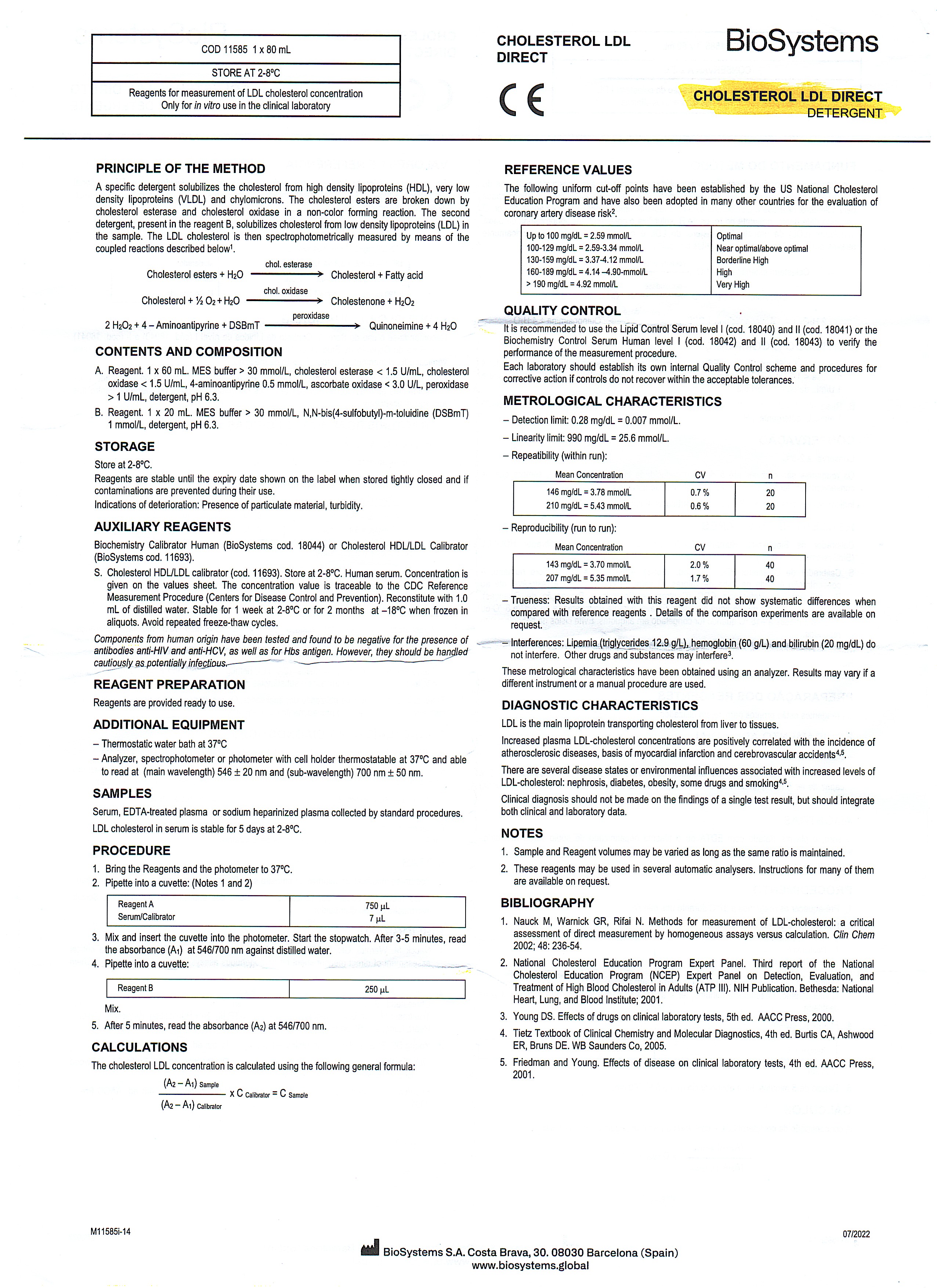

Supplement: Supplementary file 1 [file medicina-62-00008-s001.zip › Supplementary S3/Protocol - low-density lipoproteins (LDL).jpg]

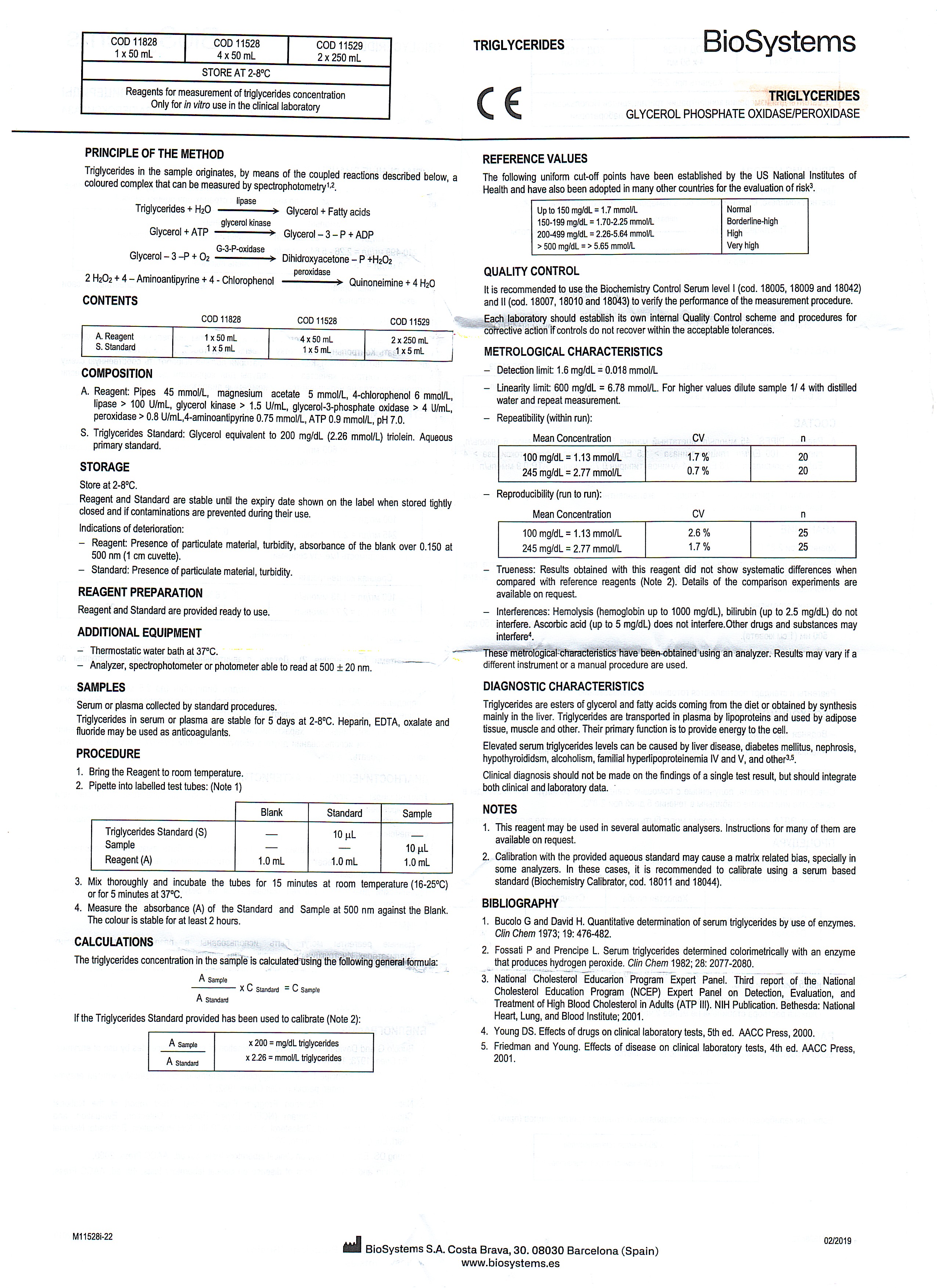

Supplement: Supplementary file 1 [file medicina-62-00008-s001.zip › Supplementary S3/Protocol - triglycerides.jpg]

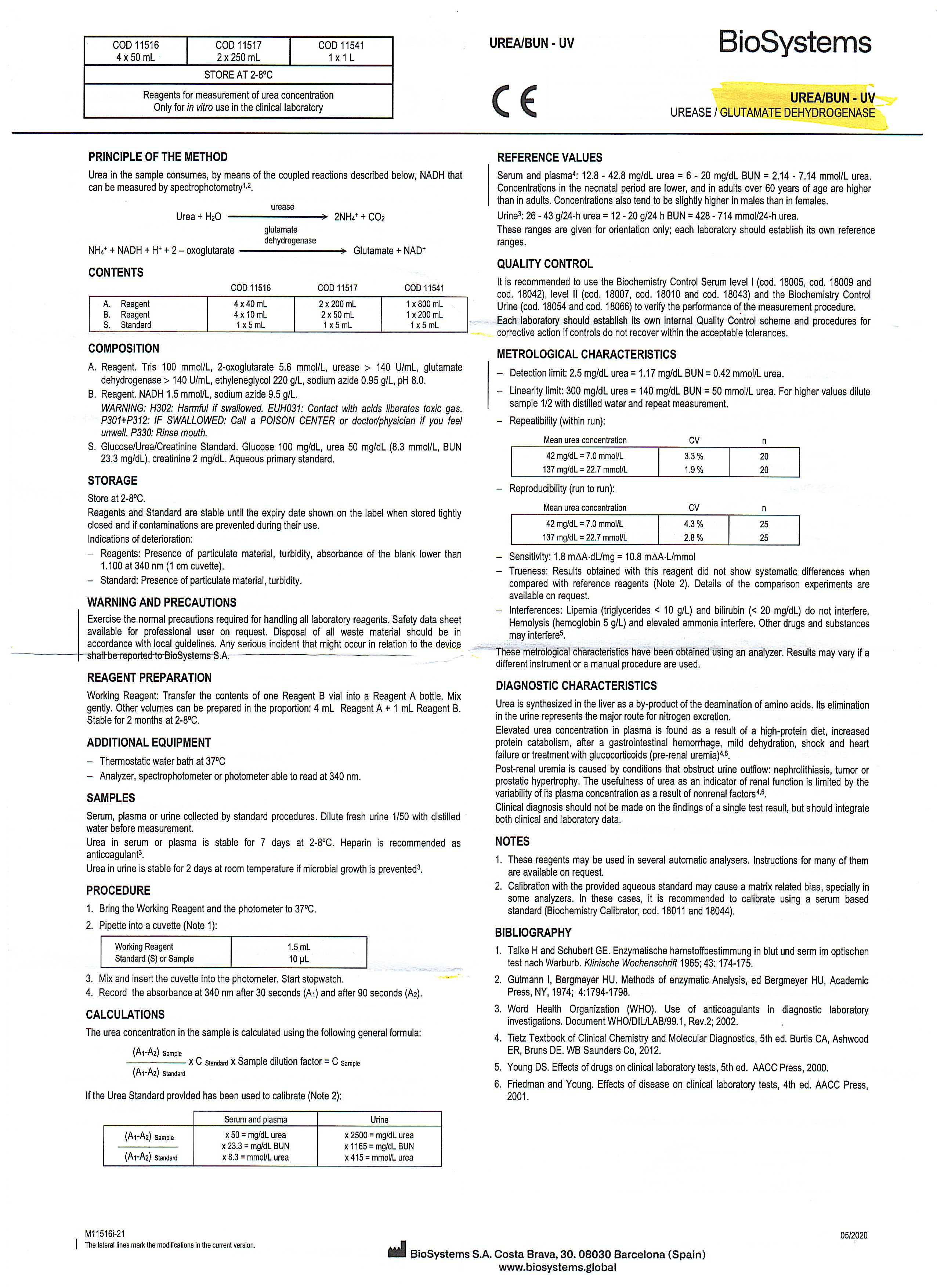

Supplement: Supplementary file 1 [file medicina-62-00008-s001.zip › Supplementary S3/Protocol - urea.jpg]
